# Risk Factors in the First 1000 Days of Life Associated With Childhood Obesity: A Systematic Review and Risk Factor Quality Assessment

**DOI:** 10.1111/obr.70025

**Published:** 2025-11-19

**Authors:** Sophia M. Blaauwendraad, Arwen S. J. Kamphuis, Francisco Javier Ruiz‐Ojeda, Marco Brandimonte‐Hernández, Eduard Flores‐Ventura, Marieke Abrahamse‐Berkeveld, Maria Carmen Collado, Janna A. van Diepen, Patricia Iozzo, Karen Knipping, Carolien A. van Loo‐Bouwman, Ángel Gil, Romy Gaillard

**Affiliations:** ^1^ The Generation R Study Group Erasmus MC, University Medical Center Rotterdam the Netherlands; ^2^ Department of Pediatrics Erasmus MC, University Medical Center Rotterdam the Netherlands; ^3^ Department of Biochemistry and Molecular Biology II, School of Pharmacy University of Granada Granada Spain; ^4^ Institute of Nutrition and Food Technology “José Mataix”, Centre of Biomedical Research, University of Granada Granada Spain; ^5^ Instituto de Investigación Biosanitaria IBS.GRANADA, Complejo Hospitalario Universitario de Granada Granada Spain; ^6^ Adipocytes and Metabolism Unit. Helmholtz Diabetes Center, Helmholtz Munich Munich Germany; ^7^ CIBEROBN (CIBER Physiopathology of Obesity and Nutrition), Instituto de Salud Carlos III Madrid Spain; ^8^ Institute of Agrochemistry and Food Technology‐National Research Council (IATA‐CSIC) Paterna Valencia Spain; ^9^ Danone Nutricia Research Utrecht The Netherlands; ^10^ Reckitt Benckiser/Mead Johnson Nutrition Nijmegen The Netherlands; ^11^ Institute of Clinical Physiology, National Research Council (CNR) Pisa Italy; ^12^ Ausnutria B.V Zwolle the Netherlands; ^13^ Yili Innovation Center Europe Wageningen the Netherlands

**Keywords:** childhood obesity, early life risk factors, infancy, preconception, pregnancy

## Abstract

**Background:**

Early‐life exposures might negatively affect fetal and infant development, predisposing children to obesity. This study aimed to systematically identify and evaluate risk factors for childhood obesity in preconception, pregnancy, and infancy, and assess their potential for future prediction and prevention strategies.

**Methods:**

This systematic review (PROSPERO, CRD42022355152) included longitudinal studies from selected electronic databases published between inception and August 17th, 2022, identifying maternal, paternal, or infant risk factors from preconception until infancy for childhood obesity between 2 and 18 years. Screening and data extraction were conducted using standardized forms. We assessed risk factor quality on modifiability and predictive power using a piloted criteria template from ILSI‐Europe‐Marker‐Validation‐Initiative.

**Findings:**

We identified 172 publications from observational and five publications from intervention studies involving *n* = 1,879,971 children from 37, predominantly high‐income, countries. Average reported childhood obesity prevalence was 11.1%. Pregnancy and infancy risk factors were mostly studied. We identified 59 potential risk factors; 23 were consistently associated. Strongest risk factors were: higher maternal prepregnancy weight (*n* = 28/31 publications with positive associations), higher gestational weight gain (*n* = 18/21), maternal smoking during pregnancy (*n* = 23/29), higher birth weight (*n* = 20/28), large‐size‐for‐gestational‐age‐at‐birth (*n* = 17/18), no breastfeeding (*n* = 20/31), and higher infant weight gain (*n* = 12/12). Level of evidence was generally moderate due to unreliable exposure measurement, short follow‐up/loss to follow‐up, and risk of confounding.

**Interpretation:**

We identified seven early‐life risk factors, which were strongly associated with childhood obesity, and can contribute to future prediction and prevention strategies. These findings support the implementation of prevention strategies targeting these risk factors from a clinical and population perspective, where possible integrated with implementation studies.

## 1 Introduction

Childhood obesity is a major public health challenge [1]. Childhood obesity rates have rapidly increased over the past decades and continue to rise worldwide [2]. Childhood obesity is related to increased risks of noncommunicable diseases, such as hypertension, Type 2 diabetes, and mental health disorders from childhood onwards, leads to a reduced quality of life, and large economic and societal burdens [3, 4, 5]. Accumulating evidence suggests that high susceptibility to obesity might already originate in early life. The Developmental Origins of Health and Disease (DOHaD) hypothesis states that in the early stages of human development, individuals are particularly vulnerable to an adverse environment [6]. During preconception, egg cell maturation and sperm production occur. After conception, fetal life and early infancy are characterized by rapid cell division, organ development, and rapid growth. These stages define the blueprint of later health and disease, and adverse maternal, paternal, and infant exposures during these critical developmental periods might predispose an individual to an increased risk of obesity in later life. Thus far, most studies and subsequent systematic reviews have focused on single early‐life risk factors for childhood obesity, such as maternal obesity [7] or exposure to endocrine disruptors and phthalates [8] or focused on selected populations [9]. However, it is well known that risk factors often cluster within families and may have accumulative adverse effects on childhood obesity development [10]. Two previous systematic reviews conducted by the same research group from over a decade ago, including observational and intervention studies, identified several risk factors for childhood overweight and obesity in the first 1000 days of life, with a specific focus on risk factors in pregnancy and infancy [11, 12]. These systematic reviews included overweight and obesity into a single outcome, measured from the age of 6 months onwards. However, from a clinical and public health perspective, especially childhood obesity from school‐age onwards is a major risk factor for comorbidities in childhood and adulthood and premature mortality in adulthood [3, 13, 14, 15]. These systematic reviews also did not specifically include the preconception period, which has increasingly been recognized as a potential critical period for childhood obesity development in the past few years, and research in this area is quickly expanding [16].

As the prevalence of childhood obesity is rising at an alarming pace across the world, an up‐to‐date systematic review across populations worldwide of potential early‐life risk factors for childhood obesity from preconception until infancy, thereby covering the full first 1000 days of life, is urgently needed to enable the development of improved prevention strategies with better early prediction of childhood obesity risk and potential novel modifiable targets for interventions at an individual and population level.

Therefore, we conducted a systematic review to first identify risk factors for childhood obesity in the preconception period, pregnancy, and infancy, thereby covering the critical first 1,000 days of life. Second, we aimed to assess the quality of potential crucial early‐life risk factors for the prediction of childhood obesity risk and as potential modifiable targets for future prevention strategies.

## 2 Methods

### 2.1 Systematic Review Protocol Development

This study was part of a large collaborative effort to perform a systematic review of risk factors in the first 1000 days of life for the development of various childhood cardiometabolic disorders. We developed a systematic review protocol to comprehensively include and evaluate individual research studies reporting on risk factors and noninvasive biomarkers during preconception, pregnancy, and infancy for the development of various child and adolescent cardiometabolic disorders. For this study, we aimed to identify longitudinal observational or intervention studies that focused on association studies or prediction studies for early‐risk factors for childhood obesity between 2 and 18 years.

Risk factors and noninvasive biomarkers of interest, hereafter also referred to as “risk factors,” included sociodemographic factors, lifestyle factors, including psychological factors, physical factors, environmental factors, pregnancy‐related factors, and noninvasive biomarkers. Noninvasive biomarkers included biomarkers obtained from saliva, fecal material, urine, hair, or cord blood; for example, metabolite or microbiome profiles, or miRNA‐expression patterns. We were interested in risk factors in mothers, fathers, and offspring obtained during the crucial periods: preconception, pregnancy, and infancy until 2 years, together covering the first 1000 days of life. The outcome of interest was self‐reported, physician, or researcher‐diagnosed obesity.

### 2.2 Information Sources, Search Strategy, Screening, and Eligibility Criteria

We registered our search strategy and systematic review protocol to PROSPERO CRD42022355152. We developed search terms for Medline, EMBASE, Web of Science, SCOPUS, and Cochrane CENTRAL (Text S1) for eligible citations published in the English language through August 17th, 2022. We included prospective and retrospective longitudinal observational studies identifying (nonmodifiable and modifiable) risk factors during preconception, pregnancy, and infancy of incident offspring outcomes of interest, and intervention studies prospectively comparing treatment effects on the outcomes of interest. For the definition of the preconception period, we followed the definitions used by the included studies. The majority of the studies defined the preconception period as the 3 months prior to conception or the period when women or couples are actively trying to conceive. We excluded cross‐sectional studies and studies among diseased populations only. Additional exclusion criteria were studies with offspring outcomes before the age of 2 years, studies reporting only intermediate phenotypes or continuous traits such as weight or BMI on a continuous scale, or studies that only assessed endpoints outside of the cardiometabolic outcomes of interest. Two independent reviewers screened at the title and abstract level. For accepted citations, two independent reviewers screened the full manuscripts. Conflicts at all screening stages were resolved by a third reviewer. Conflicts that were not resolved by the third reviewer were discussed in the full group. All screenings were conducted in the Covidence online systematic review tracking platform.

### 2.3 Data Extraction and Synthesis Methods

We developed and piloted a data extraction template for eligible manuscripts, which included manuscript information, study‐level details and design, population enrollment and characteristics, exposure and outcome ascertainment and diagnosis criteria, and age at offspring outcome assessment. We classified exposures in eight broad categories: (i) parental lifestyle factors, (ii) parental physical factors, (iii) environmental factors, (iv) pregnancy‐related factors, (v) birth anthropometrics, (vi) feeding patterns, (vii) infant anthropometrics, and (viii) cord blood biomarkers. When studies examined more than one risk factor, findings for each risk factor were assessed and presented separately. Direction of effects was harmonized between studies to present summarized results. When multiple publications on different risk factors originated from the same trial or cohort, the cohort or trial was counted only once in the total sample size calculation. The largest sample size of each cohort or trial was included. Our final sample size estimate thus reflects the number of unique individuals from unique cohorts and trials.

### 2.4 Risk of Bias Assessment for Quality and Certainty Assessment

We assessed the quality of each study using the Joanna Briggs Institute (JBI) critical appraisal tools for cohort studies, case–control studies, and randomized controlled trials (RCTs) [17]. A detailed description of how the JBI criteria were applied in our review is provided in Table S1. For cohort and case–control studies, we assessed quality based on 11 and 9 items, respectively, that evaluated population recruitment, exposure and outcome ascertainment, confounding, statistical methodology, and follow‐up. For the RCTs, we evaluated 11 items that assessed selection and allocation, intervention, administration, outcome ascertainment, follow‐up, and statistical analysis. JBI items were categorized as “Yes,” “No,” “Unclear,” or “Not applicable” following established guidelines. Any uncertainty in assessment was further discussed by the full research team until consensus was reached. Studies with overall scores of 50% or less on questions answered with “Yes,” 50% until 70% of questions answered with “Yes,” and 70% or more of questions answered with “Yes” were considered low, moderate, and high quality, respectively.

### 2.5 Quality Assessment of Risk Factors for Prediction and Prevention

For the quality assessment of the risk factors, we developed and piloted a criteria template for risk factor quality assessment based on the ILSI Europe Marker Validation Initiative [18]. This approach was previously developed by a multidisciplinary international Expert Group to set out and test criteria designed to aid the evaluation of candidate markers for their usefulness in nutrition research [18]. This marker assessment tool enables researchers to evaluate and compare different candidate markers within the same field of research to identify their relative usefulness, and allows the ranking to be modified according to the research setting and field. We defined the definitions of the ranking of the criteria in the planning stage of our systematic review with our full expert group.

Due to the large heterogeneity of the identified publications, we used a stepwise approach for the quality assessment of the risk factors. First, we took forward those risk factors we considered consistently associated with childhood obesity for quality assessment. To reduce the risk of neglecting novel, less frequently studied risk factors, we defined a consistently associated risk factor as a risk factor for which over 50% of studies reported an association in the same direction, in at least two studies of moderate or high quality [19]. Second, the early‐life risk factors, which were consistently associated with childhood obesity, were scored on their methodological aspects, reflection of the study objective, potential use for prediction, and potential for modifiability by interventions based on the ILSI Europe Marker Validation Initiative [18]. Risk factors were scored by three independent reviewers at four different levels based on the criteria: very strong, strong, medium, and low. The classification and definitions for the ranking are given in Table S2. Conflicts were discussed in the full group until consensus was reached. Briefly, methodological aspects included reproducibility, accuracy, standardization, stability, technical variation, and biological variation of the risk factor. Reflection of the study objective included that a change in the risk factor was linked with a change in the endpoint in one or more target populations. Risk factors were considered as potentially relevant for prediction based on the consistency of associations in good quality studies [20]. Risk factors scored strong if > 65% of studies reported an association in the same direction in at least five moderate or high quality studies, and very strong if > 80% of studies reported an association in the same direction in at least five moderate or high quality studies, respectively. Modifiability reflects the potential to design individual intervention strategies, and was based on two components: theoretically modifiability and proven modifiability of the risk factor based on intervention studies. A risk factor was considered strong in modifiability if it scored strong or very strong on theoretically modifiable (risk factor is modifiable on the individual level, and implementation of modifications in daily life is complicated or easy, respectively) and low on proven modifiability of the risk factor in intervention studies (no intervention studies have been conducted, or no potential effect of modification of the risk factor has been found). Very strong on modifiability was considered if a risk factor scored strong or very strong on theoretically modifiable and at least medium on intervention studies targeting the risk factor (there is some evidence of potential effect of intervention on the risk factor and outcome, but literature is controversial, or higher, respectively). We considered those risk factors which scored strong or very strong on methodological aspects, reflection of the study objective, prediction, and modifiability as the strongest risk factors for childhood obesity with the highest potential relevance for prediction and prevention strategies.

## 3 Results

### 3.1 Study Selection and Participant Characteristics

**FIGURE 1:**
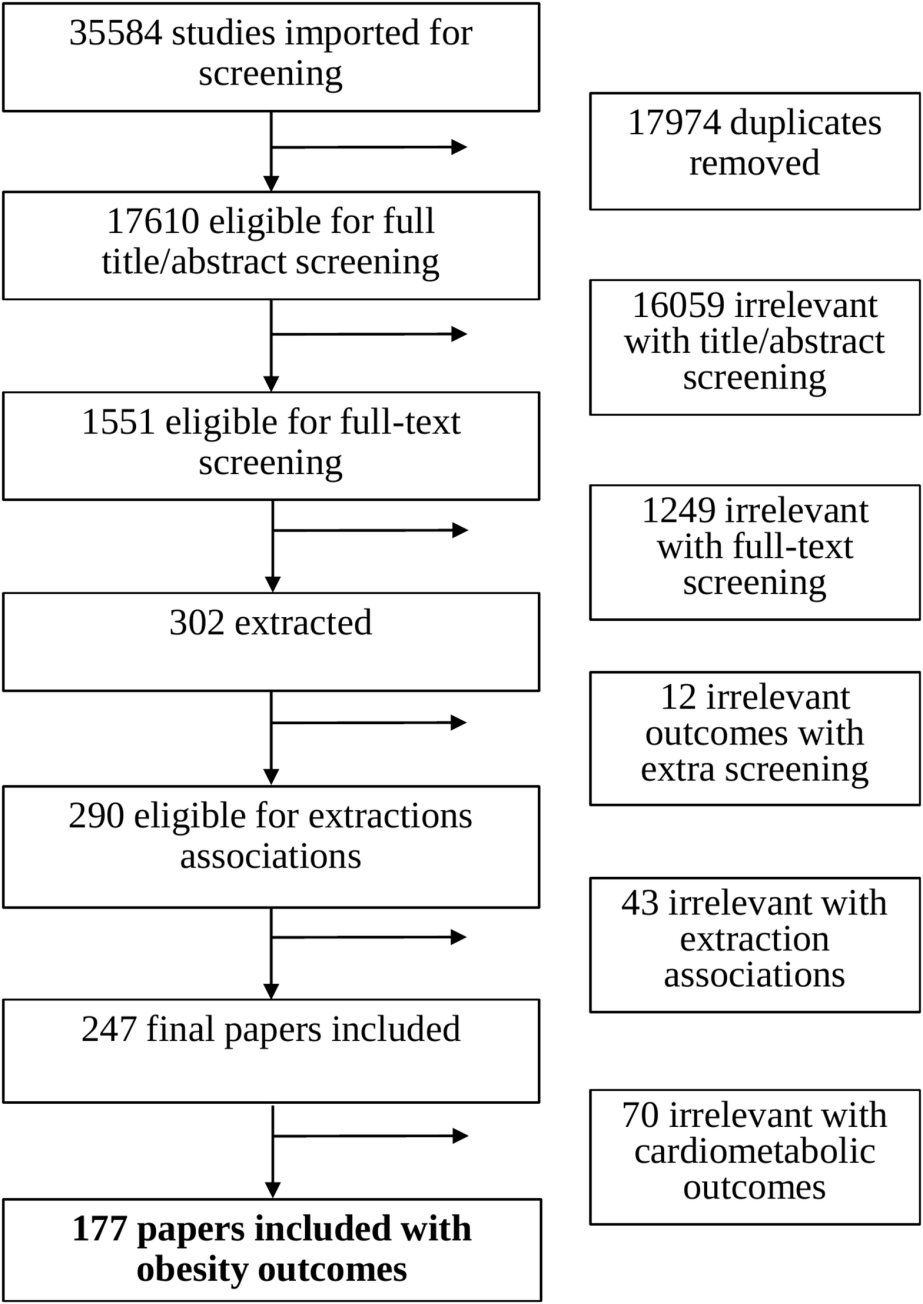
Flowchart of publications included in systematic review.

Figure 1 shows 35,584 publications were identified for screening through searches of selected electronic databases. After removing 17,974 duplicates, 17,610 publications were subjected to title and abstract screening by two independent reviewers; 16,059 publications were found to be irrelevant, and 1551 publications underwent full text screening; 177 studies met the inclusion criteria and were included in this review.

**FIGURE 2:**
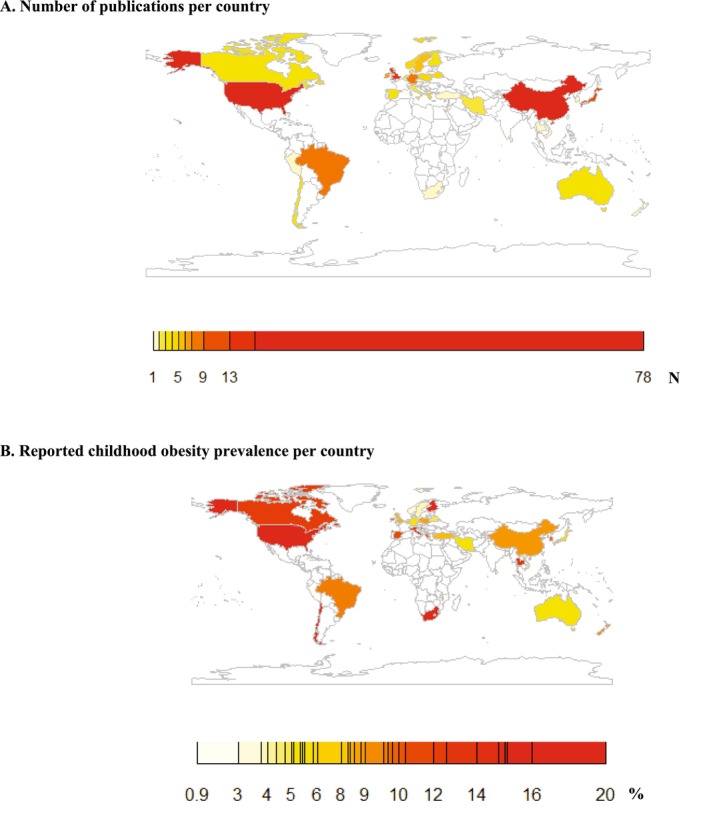
World map of the included publications. (A) Number of publications per country. (B) Reported childhood obesity prevalence per country.

Publications included 165 based on cohort studies, seven based on case–control studies, and five based on intervention studies (Table 1). In preconception, pregnancy, and infancy, 34 (100%), 135 (98.5%), and 76 (96%) were publications based on observational studies, respectively. Studies were performed between 1970 and 2022. Sample sizes varied from 50 to 155,411 participants, leading to 1,879,971 children included in the final study sample. Fifty‐nine percent of publications measured childhood obesity between 2 and 6 years, 25% between 6 and 10 years, and 16% between 10 and 18 years. The definition of childhood obesity differed between the included studies; most commonly used were the criteria according to the WHO, CDC, IOTF, and growth charts by Cole et al. The majority of publications (80%) measured obesity via measurements at research centres or medical records, 16% were based on self‐reported data, and 4% were unclear on how the outcome was measured. Figure 2A shows that publications included data from 37 different countries, predominantly high‐income countries. The average reported prevalence of obesity was 11.1%, which ranged from 0.9% in Peru to 19.6% in Finland (Figure 2B).

**TABLE 1:** Characteristics of publications included in the systematic review.

Author	Year	Country	Study type	Study name or site (Year of birth)	Exposure (time period)	Exposure (main)	Exposure (sub)	Exposure ascertainment	Definition of obesity	Sample size*
Alves	2016 [21]	Brazil	Retro. Obs.		Pregnancy and Birth	Birth anthropometrics	Adverse birth outcomes	Medical report	> 97th percentile WHO	134
Aris	2017 [22]	Singapore	Pros. Obs.	Growing up in Singapore Towards Healthy Outcomes Study	Infancy	Infant anthropometrics	BMI change	Medical report	Age‐ and sex specific BMI *z*‐scores 2 SD > median of World Health Organization Growth Charts	1170
Aris	2018 [23]	USA; Belarus	Pros. Obs.	Project Viva and Promotion of Breastfeeding Intervention Trial	Infancy	Infant anthropometrics	Weight change	Medical report	BMI ≥ 95th percentile CDC	13,666
Brophy	2009 [24]	UK	Pros. Obs.	Millenium Cohort Study	Preconception; Pregnancy and Birth; Infancy	Birth anthropometrics; Feeding patterns; Physical	Birth weight; anthropometrics; introduction to solid food	Self‐reported	International Obesity Task Force definitions	17,561
Bryl	2022 [25]	Poland	Retro. Obs.		Pregnancy and birth	Lifestyle	Maternal stress	Self‐reported	International Obesity Task Force definitions	530
Burdette	2007 [26]	USA	Pros. Obs.	The fragile families and child well‐being study	Infancy	Feeding patterns	Breastfeeding	Self‐reported	BMI > 95th percentile CDC	2146
Callanan	2021 [27]	Ireland	Intervention Study	A randomized control trial of a low glycaemic index diet in pregnancy to prevent macrosomia (ROLO)	Pregnancy and Birth	Lifestyle	Diet	Medical report	UK‐growth chart cut‐off > 98th percentile or WHO‐ cut off ≥ 95th percentile	
Carrillo‐Larco	2015 [28]	Peru	Pros. Obs.	Young Lives Study	Pregnancy and Birth	Pregnancy Complications	Mode of delivery	Self‐reported	International Obesity Task Force definitions	974
Chen	2006 [29]	USA	Pros. Obs.	Collaborative Perinatal Project (CPP)	Pregnancy and Birth	Lifestyle	Smoking	Self‐reported	Age‐ and sex‐specific BMI > 95th percentile CDC	34,866
Chen	2017 [30]	Taiwan	Pros. Obs.	Taiwan Birth Cohort Study (TBCS)	Pregnancy and Birth	Sociodemographic; Pregnancy complications; Birth anthropometrics;	Maternal age; anthropometrics; gestational weight gain; gestational diabetes mode of delivery; gestational age;	Self‐reported; medical report	BMI by Taiwan Bureau of Health Promotion for preschool children	19,269
Chen	2019a [31]	Ireland	Pros. Obs.	Lifeways Cross generation Cohort	Pregnancy and birth	Lifestyle	Diet	Self‐reported	BMI *z*‐score > 95th percentile, UK growth reference Cole	842
Chen	2019b [32]	China	Retro. Obs.		Pregnancy and birth	Birth anthropometrics	Adverse birth outcomes	Self‐reported	International Obesity Task Force definitions	62,715
Chiasson	2016 [33]	USA	Pros. Obs.	New York State Special Supplemental Nutrition Program for Women, infants, and children	Pregnancy and birth; Infancy	Birth anthropometrics; Feeding patterns	Adverse birth outcomes; breastfeeding	Self‐reported; medical report	Age‐ and sex‐ specific BMI ≥ 95th percentile CDC	50,589
Chivers	2012 [34]	Australia	Pros. Obs.	The Western Australian Pregnancy Cohort (RAINE)	Preconception; Pregnancy and birth	Sociodemographic; Lifestyle; Physical	Education; smoking; anthropometrics	Self‐reported	International Obesity Task Force definitions	1403
Dal'Maso	2022 [35]	Brazil	Retro. Obs.		Pregnancy and birth	Pregnancy complications	Mode of delivery	Self‐reported	BMI *z*‐score obesity > 2 to ≤ 3, severe obesity > 3, WHO	2405
De Sousa	2013 [36]	Brazil	Retro. Obs.		Pregnancy and birth	Birth anthropometrics	Adverse birth outcomes	Self‐reported; medical report	BMI > 95th percentile	250
Dhana	2018 [37]	USA	Pros. Obs.	Nurses' Health Study II (NSHII) and Growing Up Today Study 2 (GUTS2)	Preconception	Lifestyle; Physical	Smoking; diet; physical activity; anthropometrics	Self‐reported	According to IOTF criteria	5701
Diesel	2015a [38]	USA	Pros. Obs.	Maternal Health Practices and Child Development project	Pregnancy and birth	Physical	Gestational weight gain	Self‐reported	BMI *z*‐score ≥ 95th percentile on sex‐ and age‐adjusted growth charts by centers for Disease Control (CDC)	609
Diesel	2015b [39]	USA	Pros. Obs.		Pregnancy and birth	Physical	Gestational weight gain	Self‐reported	Age‐and sex‐adjusted BMI *z*‐scores according to the CDC growth references	514
Donahue	2011 [40]	USA	Pros. Obs.	Project Viva	Pregnancy and birth	Lifestyle; Cord blood biomarkers	Diet; lipids; fatty acids	Self‐reported	BMI *z*‐score ≥ 95th percentile on sex‐ and age‐adjusted growth charts by centers for Disease Control (CDC)	1250
Donkor	2017 [41]	Norway	Retro. Obs.		Pregnancy and birth; Infancy	Sociodemographic; Lifestyle; Birth anthropometrics; Feeding patterns	Age; smoking; birth weight; birth length; breastfeeding	Self‐reported; medical report	BMI cut‐offs according to the IOTF criteria	1895
Durmus	2011 [42]	The Netherlands	Pros. Obs.	The Generation R study	Pregnancy and birth	Lifestyle	Smoking	Self‐reported	Sex‐ and age‐adjusted BMI distribution > 2.3 SDS according to Cole et al.	5342
Ehrenthal	2016 [43]	USA	Pros. Obs.	Delaware Mother Baby Cohort	Infancy	Feeding patterns	Breastfeeding	Medical report	BMI ≥ 95th percentile for sex‐ and age CDC	2172
Eid	1970 [44]	UK	Retro. Obs.		Pregnancy and birth; Infancy	Birth anthropometrics; Infant anthropometrics	Adverse birth outcomes; weight change	Medical report	Body weight > 20% of expected weight for height and sex, according to Scotts	224
Ensenauer	2013 [45]	Germany	Retro. Obs.		Pregnancy and birth	Physical	Gestational weight gain	Self‐reported; medical report	BMI cut‐offs according to IOTF	6837
Fernandez‐Barres	2016 [46]	Spain	Pros. Obs.	Infancia Medio Ambiente Birth Cohort (INMA)	Preconception; Pregnancy and birth; Infancy	Sociodemographic; Lifestyle; Physical; Pregnancy complications Birth anthropometrics; Feeding patterns	Age; social class; education level; smoking; diet; physical activity; gestational diabetes; gestational weight gain; birth weight; breastfeeding	Self‐reported	> 95th percentile by WHO reference	1827
Fisch	1975 [47]	USA	Pros. Obs.	Collaborative Perinatal Project	Preconception; Pregnancy and birth	Sociodemographic; Lifestyle; Physical; Birth anthropometrics	Socioeconomic status age; smoking; anthropometrics; gestational weight gain; gestational age; birth weight	Medical report	Ratio index (weight/height) ≥ 95th percentile	1786
Flemming	2013 [48]	Canada	Retro. Obs.	The children's lifestyle and School Performance Study	Pregnancy and birth	Pregnancy complications	Mode of delivery	Self‐reported; medical report	BMI cut‐offs according to IOTF	2988
Flores	2013 [49]	USA	Pros. Obs.	The birth Cohort of Early Childhood Longitudinal Study (ECLS‐B)	Preconception; Pregnancy and birth	Sociodemographic; Physical; Pregnancy complications	Age; anthropometrics; gestational diabetes	Self‐reported; medical report	Age‐ and sex‐specific BMI ≥ 99th percentile CDC	6800
Frondelius	2018 [50]	Sweden	Retro. Obs.		Pregnancy and birth; Infancy	Sociodemographic; Lifestyle; Feeding patterns	Parity; smoking; breastfeeding	Self‐reported; medical report	Cut‐off for BMI according to Cole et al.	5815
Gaillard	2013 [51]	Netherlands	Pros. Obs.	The Generation R Study	Preconception	Physical	Anthropometrics	Medical reports	BMI cut‐offs according to IOTF	4571
Gallo	2016 [52]	Italy	Retro. Obs.		Pregnancy and birth	Birth anthropometrics	Adverse birth outcomes	Medical report	BMI *z*‐score > 1.7 according to CDC growth charts	7218
Gete	2021 [53]	Australia	Pros. Obs.	The Australian Longitudinal Study on Women's Health (ALSWH) and the Mothers and their Children's Health (MatCH) study	Preconception	Lifestyle	Diet	Self‐reported	According to sex‐ and age‐sepecific BMI classifications (Cole et al.)	3391
Gillman	2003 [54]	USA	Pros. Obs.	Growing up Today Study	Pregnancy and birth	Pregnancy complications; Birth anthropometrics	Gestational diabetes; Birth weight	Self‐reported	BMI>age‐ and gender‐specific 95th percentile CDC	14,881
Gittner	2013 [55]	USA	Retro. Obs.		Preconception; Pregnancy and birth; Infancy	Birth anthropometrics; Infant anthropometrics; Feeding patterns	Birth weight; BMI change; introduction to solid food; breastfeeding	Medical report	Age‐ and gender specific precentile by WHO BMI criteria	221
Goldani	2013 [56]	Brazil	Retro. Obs.		Pregnancy and birth	Pregnancy complications	Mode of delivery	Self‐reported	BMI ≥ 95th percentile according to gender and age in months	1463
Goodell	2009 [57]	USA	Retro. Obs.	Health Insurance Portability and Accountability Act‐compliant study	Pregnancy and birth; Infancy	Birth anthropometrics; Infant anthropometrics	Adverse birth outcomes; weight change	Medical report	BMI‐for‐age and sex percentile ≥ 95th percentile CDC	203
Gooze	2011 [58]	USA	Pros. Obs.	the Early Childhood Longitudinal Study, Birth Cohort	Infancy	Feeding patterns	Introduction to solid food; breastfeeding	Self‐reported	Sex‐specific BMI‐for‐age ≥ 95th percentile	6750
Groth	2017 [59]	USA	Pros. Obs.	The New Mothers study	Preconception; Pregnancy and birth	Physical	Anthropometrics; gestational weight gain	Self‐reported	Adjusted BMI percentile > 95th percentile CDC	295
Grube	2015 [60]	Germany	Retro. Obs.	German Health Interview and Examination Survey for Children and Adolescents (KiGGS baseline study)	Infancy	Feeding patterns	Breastfeeding	Self‐reported	BMI > 97th percentile of the German reference system (Kromeyer‐Hauschild et al.)	8034
Gu	2012 [61]	China	Pros. Obs.		Pregnancy and birth	Pregnancy complications	Adverse birth outcomes	Medical report	For boys BMI ≥ 19.2 and for girls ≥ 18.9	5837
Guo	2020a [62]	China	Pros. Obs.		Pregnancy and birth	Environmental	Endocrine‐disrupting chemicals exposure (PFAS, phthalates, bispehnols)	Biochemical tests	BMI *z*‐scores > 2	430
Guo	2020b [63]	China	Pros. Obs.	Sheyang Mini Birth Cohort Study (SMBCS)	Pregnancy and birth	Environmental	Polybrominated diphenyl ethers	Biochemical tests	BMI *z*‐scores > 2 according to WHO criteria	318
Hack	2014 [64]	USA	Pros. Obs.		Pregnancy and birth	Birth anthropometrics	Adverse birth outcomes	Medical report	BMI ≥ 95th percentile	263
Hakanen	2016 [65]	Finland	Pros. Obs.		Pregnancy and birth	Pregnancy complications	Gestational diabetes	Medical report	Finnish BMI cut‐off values and International Obesity Task Force definitions	6909
Hakola	2017 [66]	Finland	Pros. Obs.	Finnish type 1 Diabetes Prediciton and Prevention study	Pregnancy and birth	Lifestyle	Diet	Self‐reported	International Obesity Task Force definitions and World Health Organization (WHO)	3807
Harris	2013 [67]	USA	Pros. Obs.	Nurses' Health Study II (NHSII)	Pregnancy and birth	Lifestyle	Smoking	Self‐reported	BMI ≥ 30	35,370
Hawkins	2019 [68]	USA	Pros. Obs.	Linked the Collecting Electronic Nutrition Trajectory Data Using e‐Records of Youth (CENTURY) Study	Pregnancy and birth; Infancy	Lifestyle; Pregnancy complications; Feeding patterns	Smoking; gestational diabetes; mode of delivery; breastfeeding	Medical report	BMI ≥ 95th percentile for age and sex based on CDC	43,894
He	2000 [69]	China	Pros. Obs.		Pregnancy and birth	Birth anthropometrics	Adverse birth outcomes	Medical report	Weight that exceeded the standard weight for height, age and sex by more than 20% or, equivalently, a height‐adjusted weight over 120% of the NCHS mean	465
Heerman	2019 [70]	USA	Pros. Obs.	Growing Right Onto Wellness	Pregnancy and birth; Infancy	Birth anthropometrics; Feeding patterns;	Gestational age; birth weight; breastfeeding	Self‐reported	BMI ≥ 95th percentile for age and sex based on CDC	605
Herath	2020 [71]	Sri Lanka	Retro. Obs.		Pregnancy and birth	Pregnancy complications	Hyperglycaemia in pregnancy	Self‐reported; medical report	BMI, both IOTF and WHO reference	412
Hildebrand	2022 [72]	USA	Pros. Obs.	Eunice Kennedy Shriver National Institute of Child Health and Human Development (NICHD) Fetal Growth Studies‐Singletons cohort, and Environmental Exposures and Child Health Outcomes (ECHO) study	Infancy	Feeding patterns	Breastfeeding	Self‐ reported	BMI ≥ 95th percentile for age and sex based on CDC	823
Hinkle	2012 [73]	USA	Pros. Obs.	Early Childhood Longitudinal Study‐Birth Cohort	Preconception	Physical	Anthropometrics	Self‐reported; medical report	BMI ≥ 95th percentile for age and sex based on CDC 2000 growth charts	3600
Hivert	2016 [74]	USA	Pros. Obs.	Project Viva	Pregnancy and birth	Physical	Gestational weight gain	Self‐reported	BMI ≥ 95th percentile of the study population	979
Horiuchi	2021 [75]	Japan	Pros. Obs.	The Japan Environment and Children's Study	Pregnancy and birth	Lifestyle	Smoking	Self‐reported	BMI > 95th percentile according to the child growth standards of the WHO according to sex	24,366
Hu	2019 [76]	USA	Pros. Obs.	The Conditions Affecting Neurocognitive Development and Learning in Early Childhood (CANDLE) study	Preconception; Pregnancy and birth	Physical; Pregnancy complications	Anthropometrics; gestational weight gain; gestational diabetes	Self‐reported; medical report	BMI ≥ 95th percentile CDC	1425
Hu	2020 [77]	USA	Pros. Obs.	The Conditions Affecting Neurocognitive Development and Learning in Early Childhood (CANDLE) study	Pregnancy and birth	Lifestyle	Diet	Self‐reported	BMI 95th ≥ percentile for children of the same age and sex.	1257
Huang	2014 [78]	USA	Retro. Obs.	The 1979 National Longitudinal Survey of Youth	Preconception; Pregnancy and birth; Infancy	Sociodemographic; Lifestyle; Physical; Pregnancy complications; Birth anthropometrics; Feeding patterns	Age; ethnicity; education; employment; alcohol; smoking; anthropometrics; mode of delivery; birth weight; adverse birth outcomes; breastfeeding	Self‐reported	BMI ≥ 95th percentile for age and sex based on CDC 2000 growth charts	5156
Huh	2011 [79]	USA	Pros. Obs.	Project Viva	Infancy	Feeding pattern	Introduction to solid food	Self‐reported	BMI ≥ 95th percentile for US reference database CDC	847
Huh	2012 [80]	USA	Pros. Obs.	Project Viva	Pregnancy and birth	Pregnancy complications	Mode of delivery	Medical report	BMI for age‐ and sex ≥ 95th percentile CDC	1255
Hummel	2021 [81]	USA; Finland; Germany; Sweden	Pros. Obs.	The Environmental Determinants of Diabetes in the Young (TEDDY) study	Infancy	Feeding patterns	Breastfeeding	Self‐reported	BMI‐SDS > 2, WHO growth curves	4927
Hunt	2022 [82]	USA	Pros. Obs.	Environmental Influences on Child Health Outcomes (ECHO‐FGS)	Preconception; Pregnancy and birth; Infancy	Sociodemographic; Physical; Feeding patterns	Age; education; anthropometrics; gestational weight gain; breastfeeding	Self‐reported; medical report	BMI for age‐ and sex ≥ 95th percentile CDC	816
Huus	2007 [83]	Sweden	Pros. Obs.	All Babies in Southeast Sweden (ABIS) study	Pregnancy and birth; Infancy	Sociodemographic; Birth anthropometrics	Age; marital status; adverse birth outcomes; BMI change	Self‐reported	BMI > 30, Cole et al.	5999
Huus	2008 [84]	Sweden	Pros. Obs.	All babies in Souteast Sweden (ABIS) study	Infancy	Feeding patterns	Breastfeeding	Self‐reported	BMI cutoffs for age‐and gender according to Cole et al.	5999
Ingstrup	2012 [85]	Denmark	Pros. Obs.	The Danish national birth cohort (DNBC)	Pregnancy and birth	Maternal distress; Sociodemographic	Maternal distress and worrying; socioeconomic status	Self‐reported	BMI for age‐ and sex ≥ 95th percentile by Cole et al.	37,764
Ino	2011 [86]	Japan	Retro. Obs.		Pregnancy and birth	Lifestyle	Smoking	Self‐reported	BMI > 25 and/or degree of obesity (DO) > 30%	2508
Izadi	2013 [87]	Iran	Retro. Obs.	Childhood and Adolescence Surveillance and PreventIon of Adult Noncommunicable disease Study CASPIAN‐III	Infancy	Feeding patterns	Breastfeeding	Self‐reported	BMI > 95th percentile Adult Treatment Panel III criteria modified for children and adolescents over weight	5528
Janjua	2012 [88]	USA	Pros. Obs.		Preconception; Pregnancy and Birth	Lifestyle; Physical; Birth anthropometrics	Smoking; anthropometrics adverse birth outcomes	Self‐reported	BMI for age and sex ≥ 95th percentile CDC	740
Jing	2014 [89]	China	Pros. Obs.	The China family Panel Studies	Infancy	Feeding patterns	Breastfeeding	Self‐reported	BMI for age and sex ≥ 95th percentile CDC	7967
Jwa	2014 [90]	Japan	Pros. Obs.	The longitudinal Survey of Babies in 21st Century	Infancy	Feeding patterns	Breastfeeding; formula feeding	Self‐reported	Cut‐offs according to IOTF	41,572
Kadawathagedara	2018 [91]	Norway	Pros. Obs.	Norwegian Mother and Child Cohort Study (MoBa)	Pregnancy and birth	Lifestyle	Dietary acrylamide intake	Self‐reported	Cut‐offs according to IOTF	51,952
Kapral	2018 [92]	USA	Retro. Obs.	Early Childhood Longitudinal Survey‐Kindergarten Cohort 2011	Pregnancy and birth	Birth anthropometrics	Adverse birth outcomes	Self‐reported	BMI age‐ and sex‐specific percentiles and *z*‐scores using the Centres for Disease Control and Prevention growth charts ≥ 95th percentile	10,186
Kato	2014 [93]	Japan	Retro. Obs.		Pregnancy and birth; Infancy	Birth anthropometrics; Infant anthropometrics;	Adverse birth outcomes; weight change; height change	Self‐reported; Medical records	≥ 90th percentile based on the reference values for Japanese children and ≥ 25 (kg/m^2^), respectively	2678
Kjaer	2019 [94]	USA	Pros. Obs.		Preconception; Pregnancy and birth; Infancy	Sociodemographic; Lifestyle; Birth anthropometrics; Feeding patterns	Anthropometrics; age; education; depression; birth weight; breastfeeding	Self‐reported	BMI for age *z*‐score ≥ 95th percentile of CDC growth charts	143
Klebanoff	2015 [95]	USA	Pros. Obs.	The Collaborative Perinatal Project	Pregnancy and birth	Lifestyle	Serum paraxanthine concentriations	Biochemical tests	BMI for age *z*‐score ≥ 95th percentile of CDC growth charts	1815
LaGasse	2011 [96]	USA	Pros. Obs.	Maternal Lifestyle Study (MLS)	Preconception; Pregnancy and birth; Infancy	Sociodemographic; Physical; Birth anthropometrics; Infant anthropometrics	Alcohol consumption; drug abuse; adverse birth outcomes; weight change	Self‐reported; medical records	BMI for age *z*‐score ≥ 95th percentile of CDC growth charts	561
Lavin	2018 [97]	Vietnam	Pros. Obs.	Young Lives	Pregnancy and birth	Sociodemographic; Lifestyle; Pregnancy complications;	Parity; smoking; mode of delivery	Self‐reported	BMI relative to the World Health Organization (WHO) international growth standards, > 2 SDs above the reference median	1937
Lawrence	2016 [98]	USA	Pros. Obs.	Early Childhood Longitudinal Study‐Birth Cohort (ECLS‐B)	Pregnancy and birth	Birth anthropometrics	Adverse birth outcomes	Self‐reported; Medical records	BMI for age *z*‐score ≥ 95th percentile of CDC growth charts	3700
Layte	2014 [99]	Ireland	Pros. Obs.	The Child Benefit Register for the Republic of Ireland	Pregnancy and birth; Infancy	Sociodemographic; Lifestyle; Physical; Birth anthropometrics; Feeding patterns	Age; social class; parity; ethnicity; alcohol consumption; smoking; birth weight; adverse birth outcomes; gestational weight gain; breastfeeding	Self‐reported; medical records	International Obesity Task Force definitions	9057
Leonard	2017 [100]	USA	Pros. Obs.	National Longitudinal Survey of Youth 1979 (NLSY79), Children and Young Adults (NLSY79‐CYA) subcohort	Preconception	Physical	Anthropometrics	Medical reports	BMI for age *z*‐score ≥ 95th percentile of CDC growth charts	4436
Li	2011 [101]	USA	Pros. Obs.	Early Childhood Longitudinal Study‐Birth Cohort (ECLS‐B)	Preconception; Pregnancy and birth; Infancy	Sociodemographic; Physical; Birth anthropometrics; Infant anthropometrics; Feeding patterns	Anthropometrics; gestational weight gain; adverse birth outcomes; breastfeeding; weight gain	Self‐reported; medical records	BMI for age *z*‐score ≥ 95th percentile of CDC growth charts	8150
Li	2015 [102]	USA	Pros. Obs.	Kaiser Permanente Northern California study	Pregnancy and birth	Lifestyle	Caffeine consumption	Self‐reported	BMI for age *z*‐score ≥ 95th percentile of CDC growth charts	801
Li	2020 [103]	USA	Pros. Obs.	Kaiser Permanente Northern California study	Pregnancy and birth	Pregnancy complications	Maternal infection; prenatal antibiotics	Medical records	BMI for age *z*‐score ≥ 95th percentile of CDC growth charts	145,393
Liu	2017 [104]	USA	Pros. Obs.	The Infant Feeding Practices Study II	Infancy	Infant anthropometrics	BMI change	Self‐reported; medical records	BMI for age *z*‐score ≥ 95th percentile of CDC 2000 growth charts	1169
Liu	2019 [105]	USA	Pros. Obs.		Pregnancy and birth	Physical	Gestational weight gain	Self‐reported	BMI for age *z*‐score ≥ 95th percentile of CDC 2000 growth charts	1296
Loaiza	2011 [106]	Chile	Pros. Obs.		Pregnancy and birth	Sociodemographic; Birth anthropometrics	Education; adverse birth outcomes	Self‐reported	BMI for age *z*‐score ≥ 95th percentile of CDC growth charts	119,070
Lowe	2018 [107]	USA	Pros. Obs.	Hyperglycemia and Adverse Pregnancy Outcome Follow Up Study (HAPO FUS)	Pregnancy and birth	Pregnancy complications	Gestational diabetes	Biochemical tests	International Obesity Task Force (IOTF) definitions	4832
Lowe	2019 [108]	Thailand; Barbados; USA; UK; China; Israel; Canada	Pros. Obs.	Hyperglycemia and Adverse Pregnancy Outcome Follow Up Study (HAPO FUS)	Pregnancy and birth	Pregnancy complications	Gestational diabetes	Biochemical tests	International Obesity Task Force definitions	4832
Mardones	2008 [109]	Chile	Pros. Obs.		Pregnancy and birth	Sociodemographic; Birth anthropometrics	Education; gestational age at birth; birth weight; birth length		BMI for age *z*‐score ≥ 95th percentile of CDC growth charts	153,536
Mardones	2014 [110]	Chile	Retro. Obs.		Pregnancy and birth	Birth anthropometrics	Birth weight; birth length	Self‐reported	BMI ≥ 95th percentile CDC	3290
Margetaki	2022 [111]	Greece	Pros. Obs.	The RHEA study	Pregnancy and birth	Pregnancy complications	Prenatal antibiotics	Self‐reported	International Obesity Task Force definitions	747
Martin	2013 [112]	Belarus	Intervention Study	Promotion of Breastfeeding Intervention Trial	Infancy	Feeding patterns	Breastfeeding	Self‐reported	BMI for age *z*‐score ≥ 95th percentile of CDC 2000 growth charts	13,879
Martin	2017 [113]	Belarus	Intervention Study	Promotion of Breastfeeding Intervention Trial	Infancy	Feeding patterns	Breastfeeding	Self‐reported	BMI for age *z*‐score ≥ 95th percentile of CDC 2000 growth charts	13,557
Masukume	2019 [114]	New Zealand	Pros. Obs.	Growing Up in New Zealand cohort	Pregnancy and birth	Pregnancy complications	Mode of delivery	Medical records	International Obesity Task Force definitions	6599
Mayer‐Davis	2006 [115]	USA	Retro. Obs.	Growing Up Today Study	Infancy	Feeding patterns	Breastfeeding; formula feeding	Self‐reported	BMI for age *z*‐score ≥ 95th percentile of CDC growth charts	15,253
McCrory	2012 [116]	Ireland	Retro. Obs.	Growing Up in Ireland Study	Pregnancy and birth; Infancy	Birth anthropometrics; Feeding patterns	Gestational age; breastfeeding	Self‐reported	International Obesity Task Force definitions	7798
Mehta	2012 [117]	USA	Retro. Obs.		Preconception; Pregnancy and birth	Physical; Pregnancy complications; Birth anthropometrics	Anthropometrics; gestational diabetes; adverse birth outcomes	Medical records	BMI ≥ 95th percentile CDC	493
Metzger	2010 [118]	USA	Pros. Obs.		Infancy	Feeding patterns	Breastfeeding	Self‐reported	BMI ≥ 95th percentile CDC	976
Michels	2007 [119]	USA	Pros. Obs.	Nurses' Mothers' Cohort Study	Infancy	Feeding patterns	Breastfeeding	Self‐reported	BMI ≥ 30 kg/m^2^	35,526
Mizutani	2007 [120]	Japan	Pros. Obs.	Project Enzan	Pregnancy and birth	Lifestyle	Smoking; diet; physical activity; sleep	Self‐reported; medical records	Definition used from Poskitt et al. (European Obesity Group)	1417
Monteiro	2003 [121]	Brazil	Pros. Obs.		Pregnancy and birth; Infancy	Birth anthropometrics; Infant anthropometrics;	Birth weight; adverse birth outcomes; weight change; height change; BMI change; skinfolds	Medical records; Non‐specified	Obesity was defined by the presence of overweight in 1997 plus both tricipital and subscapular skinfolds 90th percentile of NHANES I, as assessed in 1998.	1076
Morovic	2019 [122]	Croatia	Retro. Obs.	WHO Europe Childhood Obesity Surveillance Initiative	Infancy	Feeding patterns	Breastfeeding	Self‐reported	WHO criteria	5662
Moss	2014 [123]	USA	Pros. Obs.		Infancy	Feeding patterns	Introduction to solid food; breastfeeding	Self‐reported	BMI ≥ 95th percentile CDC	7200
Mueller	2015 [124]	USA	Pros. Obs.	Columbia Center for Children's Environmental Health Mothers and Newborn Study	Pregnancy and birth	Pregnancy complications	Mode of delivery; prenatal antibiotics	Medical records	BMI ≥ 95th percentile CDC	436
Navarro	2020 [125]	Ireland	Pros. Obs.	Lifeways Cross‐Generation Cohort Study	Preconception; Pregnancy and birth	Lifestyle; Physical	Alcohol consumption; Smoking; diet; physical activity; anthropometrics	Self‐reported	International Obesity Task Force definitions	585
Nehring	2013 [126]	Germany	Retro. Obs.	German Perinatal Prevention of Obesity cohort	Pregnancy and birth	Pregnancy complications	Gestational diabetes	Medical records; biochemical tests	International Obesity Task Force definitions	7355
Noda	2022 [127]	Japan	Pros. Obs.		Preconception	Lifestyle	Physical activity	Self‐reported	BMI ≥ 95th percentile CDC reference Japanese children	65,245
Ochoa	2007 [128]	Spain	Retro. Obs.		Pregnancy and birth; Infancy	Birth anthropometrics; Feeding patterns	Adverse birth outcomes; breastfeeding	Self‐reported	BMI > 97th percentile Spanish reference data	370
O'Connor	2020 [129]	USA	Pros. Obs.	Family Life Project	Preconception; Pregnancy and birth; Infancy	Lifestyle; Physical; Birth anthropometrics; Infant anthropometrics; Feeding patterns	Smoking; birth weight; anthropometrics; gestational age at birth; smoking birth weight; breastfeeding	Self‐reported; medical records	BMI ≥ 95th percentile CDC	1164
Oken	2007 [130]	USA	Pros. Obs.	Project Viva	Pregnancy and birth	Physical	Gestational weight gain	Self‐reported	BMI ≥ 95th percentile CDC with US national reference population	1044
Oken	2008 [131]	USA	Pros. Obs.	Growing Up Today Study cohort	Pregnancy and birth	Physical	Gestational weight gain	Self‐reported	BMI ≥ 95th percentile CDC	11,994
Oken	2009 [132]	USA	Pros. Obs.	Project Viva	Pregnancy and birth	Physical	Gestational weight gain	Self‐reported	BMI ≥ 95th percentile CDC	2012
Ouyang	2016 [133]	USA	Pros. Obs.	US Collaborative Perinatal Project	Preconception; Pregnancy and birth	Physical; Pregnancy complications	Gestational weight gain; placental weight; gestational diabetes; adverse birth outcomes.	Self‐reported; medical records	BMI ≥ 95th percentile CDC	154,590
Palma dos Reis	2022 [134]	Portugal	Pros. Obs.	Generation XXI Birth Cohort	Preconception; Pregnancy and birth	Pregnancy complications; Physical; Lifestyle	Pre‐eclampsia; anthropometrics; smoking	Self‐reported; medical report	According to the WHO criteria: class 1 (BMI 30–35), class 2 (BMI 35–40), Class 3 (BMI > 40)	5133
Pan	2019 [135]	China	Retro. Obs.		Pregnancy and birth	Birth anthropometrics	Adverse birth outcomes	Medical records	Weight‐for‐length/height Z score ≥ 95 percentiles using WHO growth charts	1767
Park	2015 [136]	South Korea	Retro. Obs.		Infancy	Feeding patterns	Breastfeeding	Self‐reported	(1) BMI values ≥ 95th percentile using an age‐ and gender‐specific reference growth chart for Korean children, (2) children with BMI > 25 kg/m^2^, (3) based on percent body fat, children with ≥ 20% body fat for boys and ≥ 28% body fat for girls	528
Parker	2012 [137]	USA	Pros. Obs.	Project Viva	Pregnancy and birth	Pregnancy complications; Birth anthropometrics	Fetal growth; birth weight	Medical records	BMI ≥ 95th percentile CDC	438
Pattison	2019 [138]	USA	Pros. Obs.	First Baby Study	Infancy	Feeding patterns	Breastfeeding	Self‐reported	BMI ≥ 95th percentile CDC	1626
Pei	2014 [139]	Germany	Pros. Obs.	Influences of Lifestyle‐Related Factors on the Immune System and the Development of Allergies in Childhood plus Air Pollution and Genetics (LISAplus)	Pregnancy and birth	Pregnancy complications	Mode of delivery	Self‐reported	BMI ≥ 95th percentile for age and sex using World Health Organization reference data	1734
Pettitt	1983 [140]	USA	Pros. Obs.		Pregnancy and birth	Pregnancy complications	Gestational diabetes	Medical records	At least 140% of their desirable weight	1935
Pettitt	1998 [141]	USA	Pros. Obs.		Pregnancy and birth	Pregnancy complications	Gestational diabetes	Self‐reported	Not given	595
Pitchika	2018 [142]	USA, Finland, Germany, Sweden	Pros. Obs.	The Environmental Determinants of Diabetes in the Young (TEDDY)	Pregnancy and birth	Pregnancy complications	Gestational diabetes	Self‐reported	BMI SDS > 2 according to WHO recommendations	5324
Power	2002 [143]	UK	Pros. Obs.		Pregnancy and birth	Lifestyle	Smoking	Self‐reported	BMI ≥ 90th percentile in study sample	5839
Ralphs	2021 [144]	UK	Pros. Obs.	Born in Bradford (BiB)	Pregnancy and birth	Pregnancy complications	Mode of delivery	Medical records	BMI ≥ 95th percentile by UK growth charts	6410
Reilly	2005 [145]	UK	Pros. Obs.	Avon Longitudinal Study of Parents and Children (ALSPAC)	Preconception; Pregnancy and birth; Infancy	Lifestyle; Physical; Birth anthropometrics; Feeding patterns	Smoking; anthropometrics; birth weight; weight change; introduction to solid food; breastfeeding	Self‐reported; medical records	BMI ≥ 95th percentile (UK reference data)	7758
Reynolds	2014 [146]	Ireland	Retro. Obs.	Growing Up in Ireland Study	Pregnancy and birth; Infancy	Lifestyle; Feeding patterns	Smoking; adverse birth outcomes; breastfeeding	Self‐reported	BMI ≥ 95th percentile by Cole et al.	8357
Rifas‐Shiman	2021 [147]	Belarus	Intervention Study	Promotion of Breastfeeding Intervention Trial	Pregnancy and birth	Pregnancy complications	Mode of delivery	Self‐reported	BMI ≥ 95th CDC	15,069
Rooney	2011 [148]	USA	Pros. Obs.		Pregnancy and birth; Infancy	Lifestyle; Physical; Birth anthropometrics; Infant anthropometrics	Smoking; gestational weight gain; mode of delivery; adverse birth outcomes; weight change	Medical records	CDC BMI ≥ 85th percentile	532
Rotevatn	2021 [149]	Denmark	Pros. Obs.		Preconception; Pregnancy and birth; Infancy	Sociodemographic; Environmental; Physical; Birth anthropometrics; Infant anthropometrics	Education; income; parity; anthropometrics; smoking; mode of delivery; adverse birth outcomes; weight change	Medical records	International Obesity Task Force definitions	55,041
Roy	2015 [150]	USA	Pros. Obs.	A Study of the Genetic Causes of Complex Pediatric Disorders at the Children's Hospital of Philadelphia	Pregnancy and birth; Infancy	Pregnancy complications; Birth anthropometrics Infant anthropometrics	Gestational diabetes; birth weight; BMI change	Medical records	CDC BMI *z*‐score ≥ 95th percentile	2114
Sakurai	2022 [151]	Japan	Pros. Obs.	The Japan Environment and Children's Study (JECS)	Pregnancy and birth	Pregnancy complications	Prenatal antibiotics	Self‐reported	BMI‐for‐age ≥ 95th percentile according to Japanese growth charts	56,416
Sartorius	2020 [152]	South Africa	Retro. Obs.	The South African National Income Dynamics Study (NA‐NIDS)	Pregnancy and birth	Birth anthropometrics	Adverse birth outcomes		Weight‐for‐length *z*‐score BMI‐for‐age *z*‐score of > SDS according to WHO growth charts	4710
Seipel	2013 [153]	USA	Registry Study	National Longitudinal Survey of Youth	Pregnancy and birth; Infancy	Sociodemographic; Lifestyle; Physical; Birth anthropometrics; Feeding patterns	Age; education; alcohol consumption; smoking; drug abuse; gestational weight gain; mode of delivery; adverse birth outcomes; breastfeeding	Self‐reported; medical records	CDC BMI ≥ 95th percentile	6634
Shankaran	2010 [154]	USA	Pros. Obs.	Maternal Lifestyle Study (MLS)	Pregnancy and birth	Lifestyle; Physical; Pregnancy complications; Birth anthropometrics	Ethnicity; education; smoking; alcohol consumption; drug use; anthropometrics; birth weight; adverse birth outcomes	Non‐specified	CDC BMI ≥ 95th percentile	880
Sharma	2008 [155]	USA	Registry Study	Centers for Disease Control and Prevention's Pregnancy Nutrition Surveillance System (PNSS) and Pediatric Nutrition Surveillance System (PedNSS)	Preconception; Pregnancy and birth	Lifestyle	Smoking	Self‐reported; medical records	BMI ≥ 95th percentile CDC	155,411
Shehadeh	2008 [156]	Israel	Retro. Obs.		Pregnancy and birth; Infancy	Birth anthropometrics; Feeding patterns	Birth weight; birth length; breastfeeding	Medical records	BMI ≥ 95th percentile	302
Shi	2013 [157]	Canada	Retro. Obs.	Canadian Health Measures Survey (2007–2009) (CHMS‐cycle 1)	Pregnancy and birth; Infancy	Lifestyle; Birth anthropometrics; Feeding patterns	Smoking; birth weight; breastfeeding	Self‐reported; medical records	According to WHO, BMI *z*‐score > 2 SD above the mean (≥ 97.7th percentile)	986
Shields	2006 [158]	Australia	Pros. Obs.	Mater‐University Study of Pregnancy cohort	Infancy	Feeding patterns	Breastfeeding	Self‐reported	BMI ≥ 95th percentile by Cole et al. (UK growth charts)	3698
Si	2022 [159]	China	Pros. Obs.		Pregnancy and birth	Pregnancy complications	Mode of delivery	Medical records	Weight‐for‐height > 3 SD above WHO Child Growth Standards median	10,418
Simpson	2017 [160]	UK	Pros. Obs.	Avon Longitudinal Study of Parents and Children (ALSPAC)	Pregnancy and birth	Cord blood biomarkers; Birth anthropometrics	Lipids; birth weight	Medical records; biochemical tests	International Obesity Task Force definitions by UK 1990 British growth reference	2775
Sitarik	2020 [161]	USA	Pros. Obs.	Wayne County Health Environment Allergy and Asthma Longitudinal Study (WHEALS)	Pregnancy and birth	Pregnancy complications	Mode of delivery	Medical records	CDC BMI ≥ 95th percentile	570
Skledar	2015 [162]	Croatia	Pros. Obs.		Pregnancy and birth; Infancy	Birth anthropometrics; Feeding patterns	Birth weight; birth length; breastfeeding	Self‐reported; medical records	≥ 95th percentile BMI‐for‐age growth charts CDC and Croatian Society for Pediatric Endocrinology	302
Smego	2017 [163]	USA	Pros. Obs.		Infancy	Infant anthropometrics	Weight change	Medical records	CDC BMI ≥ 95th percentile	1236
Sorrow	2019 [164]	USA	Pros. Obs.		Pregnancy and birth	Cord blood biomarkers	Metabolomics	Medical records	Weight‐for‐height *z*‐scores ≥ 95th percentile CDC	50
Suzuki	2009 [165]	Japan	Pros. Obs.	Project Koshu	Pregnancy and birth	Lifestyle	Smoking; diet; sleep	Self‐reported	Childhood obesity definition as in Cole 2000	1302
Tambalis	2018 [166]	Greece	Retro. Obs.		Infancy	Feeding patterns	Breastfeeding	Self‐reported	International Obesity Task Force definitions	5125
Taveras	2009 [167]	USA	Pros. Obs.	Project Viva	Pregnancy and birth; Infancy	Birth anthropometrics; Infant anthropometrics	Birth weight; birth length; weight change; height change; BMI change	Medical records	BMI ≥ 95th percentile CDC	559
Taveras	2011 [168]	USA	Registry Study		Infancy	Infant anthropometrics	Weight change	Medical records	BMI for age *z*‐score ≥ 95th percentile CDC	122,214
Thaware	2015 [169]	UK	Pros. Obs.	Hyperglycemia and Adverse Pregnancy Outcome (HAPO) study	Pregnancy and birth	Pregnancy complications;	Gestational diabetes	Medical records; biochemical tests	BMI *z*‐score ≥ 95th percentile	1320
Thaware	2018 [170]	UK	Pros. Obs.	Hyperglycemia and Adverse Pregnancy Outcome (HAPO) study	Pregnancy and birth	Physical	Lipids	Medical records	BMI *z*‐score ≥ 95th percentile	818
Toschke	2002 [171]	Czech Republic	Registry Study		Pregnancy and birth; Infancy	Birth anthropometrics; Feeding patterns	Adverse birth outcomes; breastfeeding	Self‐reported	BMI for age *z*‐score > 97th percentile based on the study population sample	33,768
Toschke	2007 [172]	UK	Pros. Obs.	Avon Longitudinal Study of Parents and Children (ALSPAC)	Infancy	Feeding patterns	Breastfeeding	Self‐reported	International Obesity Task Force definitions	4325
Turner	2021 [173]	UK	Retro. Obs.	Aberdeen Maternity and Neonatal Databank (AMND) and Study of Trends in Obesity in North East Scotland (STONES)	Pregnancy and birth	Pregnancy complications; Birth anthropometrics	Fetal growth; birth weight	Medical records	International Obesity Task Force definitions	4721
Vafeiadi	2015 [174]	Greece	Pros. Obs.	The RHEA study	Pregnancy and birth	Environmental	Persistent organic pollutants exposure	Biochemical tests	International Obesity Task Force definitions	689
Vafeiadi	2016 [175]	Greece	Pros. Obs.	The RHEA study	Pregnancy and birth	Environmental	Endocrine‐disrupting chemical exposure	Biochemical tests	International Obesity Task Force definitions	500
Van Rossem	2011 [176]	USA	Pros. Obs.	Project Viva	Infancy	Feeding patterns	Breastfeeding	Self‐reported	BMI ≥ 95th percentile CDC	884
Vehapoglu	2017 [177]	Turkey	Pros. Obs.		Pregnancy and birth	Birth anthropometrics	Birth weight	Self‐reported	BMI ≥ 95th percentile according to Cole et al.	4990
Ventura	2020 [178]	USA	Pros. Obs.	Infant Feeding Practices Study II (IFPS II) and Year 6 Follow‐Up (Y6FU)	Preconception; Pregnancy and birth; Infancy	Physical; Sociodemographic; Birth anthropometrics; Infant anthropometrics; Feeding patterns	Prepegnancy BMI; age; anthropometrics; ethnicity; education; marital status; income; parity; birth weight; gestational age; introduction to solid food	Self‐reported	BMI for age *z*‐score ≥ 95th percentile CDC	1062
Von Kries	2002 [179]	Germany	Retro. Obs.		Pregnancy and birth; Infancy	Lifestyle; Birth anthropometrics; Feeding patterns	Smoking; birth weight; introduction to solid food; breastfeeding	Self‐reported	BMI > 97th percentile based on European Childhood Obesity group with German reference values	6483
Wallby	2017 [180]	Sweden	Registry study		Pregnancy and birth; Infancy	Sociodemographic; Physical; Feeding patterns	Age; education; parity anthropometrics; breastfeeding	Medical reports	Defined according to Cole et al.	30,508
Wang	2014 [181]	USA	Pros. Obs.	National Institute of Child Health and Human Development Study (NICHD)	Pregnancy and birth	Lifestyle	Smoking	Self‐reported	BMI *z*‐score ≥ 95th percentile CDC	932
Wang	2017 [182]	USA	Pros. Obs.	National Institute of Child Health and Human Development (NICHD) Study of Early Child Care and Youth Development (SECCYD)	Infancy	Feeding patterns	Breastfeeding	Medical reports	BMI *z*‐score ≥ 95th percentile CDC	1234
Wang	2018 [183]	USA	Pros. Obs.	US Collaborative Perinatal Project	Pregnancy and birth	Pregnancy complications	Prenatal antibiotics	Self‐reported	BMI *z*‐score ≥ 95th percentile CDC	39,615
Wang	2022 [184]	China	Retro. Obs.		Preconception; Pregnancy and birth	Birth anthropometrics; Physical	Prepregnancy BMI; gestational weight gain; birth weight	Self‐reported	For children below 5 BMI *z*‐score > 3SD of WHO growth standards; for children above 5 BMI *z*‐score > 2SD than reference median; IOTF criteria for children 2‐18 years, obesity as over BMI of 30 kg/m^2^; China criteria obesity as BMI over 28 kg/m^2^	9501
Warner	2013 [185]	USA	Pros. Obs.	The Health Assessment of Mothers and Children of Salinas (CHAMACOS) study	Pregnancy and birth	Environmental	Pesticide exposure	Biochemical tests	BMI ≥ 95th percentile CDC	270
Weber	2014 [186]	Belgium Germany Italy Poland Spain	Intervention study	Childhood Obesity Project	Infancy	Feeding patterns	Formula feeding	Medical records	International Obesity Task Force definitions	588
Wen	2022 [187]	USA	Pros. Obs.	Infant Feeding Practices Study II	Pregnancy and birth	Sociodemographic	Education; income	Self‐reported	BMI for age *z*‐score ≥ 95th percentile CDC	1211
Whitaker	1998 [188]	USA	Pros. Obs.		Preconception; Pregnancy and birth	Physical; Pregnancy complications;	Anthropometrics; gestational diabetes	Medical records	BMI *z*‐scores ≥ 85th percentile	524
Widen	2016 [189]	USA	Pros. Obs.	Colombia Center for Children's Environmental Health Mothers and Newborns Study	Preconception; Pregnancy and birth	Physical	Anthropometrics; gestational weight gain	Self‐reported	BMI for age *z*‐score ≥ 95th percentile CDC	323
Wojcicki	2015 [190]	USA	Retro. Obs.	Pregnancy Risk Assessment Monitoring System	Preconception; Pregnancy and birth; Infancy	Sociodemographic; Physical; Lifestyle Feeding patterns	Age; ethnicity; education; income; smoking; marital status; anthropometrics; breastfeeding	Self‐reported	BMI ≥ 95th percentile (age and sex‐specific)	205
Wright	2009 [191]	USA	Pros. Obs.	Project Viva	Pregnancy and birth	Pregnancy complications	Gestational diabetes	Medical records	BMI > 95th percentile CDC	1238
Wroblewska‐Seniuk	2009 [192]	Poland	Pros. Obs.		Pregnancy and birth	Pregnancy complication	Gestational diabetes	Self‐reported	BMI ≥ 95th percentile for gender and age based on Polish reference growth charts	185
Yamakawa	2013 [193]	Japan	Pros. Obs.	Longitudinal Survey of Babies in the 21st Century	Infancy	Feeding Patterns	Breastfeeding	Self‐reported	International Obesity Task Force definitions	30,780
Yuan	2016 [194]	USA	Pros. Obs.	the Growing Up Today Study	Pregnancy and birth	Pregnancy complications	Mode of delivery	Self‐reported	International Obesity Task Force definitions	22,068
Zarrati	2013 [195]	Iran	Retro. Obs.		Pregnancy and birth	Birth anthropometrics	Birth weight; adverse birth outcomes	Self‐reported	Age‐ and sex‐specific BMI > 95th percentile CDC	1184
Zhang	2022 [196]	China	Registry study	Tianjin mother–child cohort	Preconception	Physical	Prepregnancy weight	Medical records	BMI ≥ 95th percentile of WHO growth standards	47,709
Zhou	2011 [197]	China	Retro. Obs.		Pregnancy and birth; Infancy	Pregnancy complication; Birth anthropometrics; Lifestyle; Feeding Patterns;	Prenatal music exposure; mode of delivery; birth weight; introduction to solid food	Self‐reported	International Obesity Task Force definitions	162

### 3.2 Risk Factors for Childhood Obesity in Preconception

**FIGURE 3:**
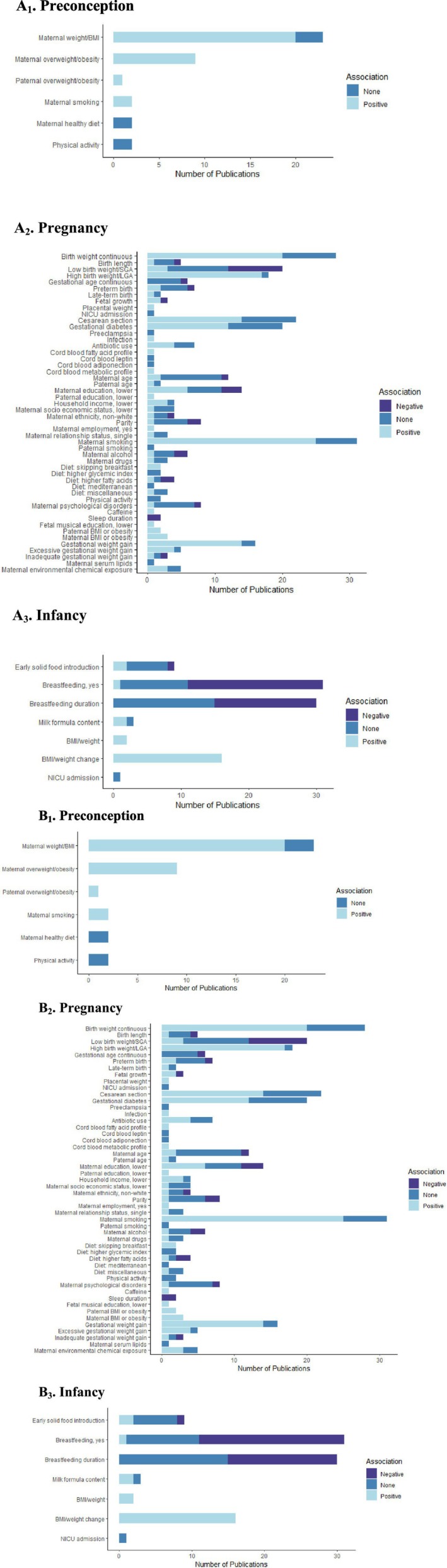
Overview of the identified early‐life risk factors and the number of reported associations with childhood obesity. (A) Figure represents reported number of positive, negative, and null associations for each identified risk factor in all included publications. A positive association indicates a significant increased risk for childhood obesity, a negative association indicates a significant decreased risk for childhood obesity and a null association indicates no significant association. When studies examined more than one risk factor, findings for each risk factor were assessed and presented separately. For presentation purposes, directions of effects were harmonized between studies to present summarized results. Maternal psychological factors are harmonized under the name of “maternal psychological disorders.” (B) Figure represents reported number of positive, negative, and null associations for each identified risk factor in only moderate to high quality publications. Low quality publications were excluded. A positive association indicates a significant increased risk for childhood obesity, a negative association indicates a significant decreased risk for childhood obesity and a null association indicates no significant association. When studies examined more than one risk factor, findings for each risk factor were assessed and presented separately. For presentation purposes, directions of effects were harmonized between studies to present summarized results. Maternal psychological factors are harmonized under the name of “maternal psychological disorders.”

We identified 34 publications from observational studies and no RCTs that assessed six risk factors associated with childhood obesity in the preconception period (Figure 3A_1_,B_1_, Table S3). Maternal physical factors were most frequently studied. Nineteen publications [24, 34, 37, 46, 47, 55, 59, 82, 94, 96, 101, 117, 129, 133, 134, 149, 184, 189, 196] based on observational studies reported that higher maternal preconception weight was associated with an increased risk of childhood obesity, whereas three publications based on observational studies [78, 178, 190] found no association. Sample sizes varied from 205 to 5156. All identified studies showed that maternal prepregnancy overweight or obesity was associated with a higher risk of childhood obesity. Only one study [145] reported paternal preconception overweight or obesity as a risk factor for childhood obesity. Four publications based on observational studies [37, 53, 127, 155] focused on the associations of maternal lifestyle factors during preconception with childhood obesity risk. Two observational studies [37, 155] showed that maternal smoking in the preconception period was associated with an increased risk of childhood obesity, whereas no associations with the risk of childhood obesity were reported for maternal preconception diet [37, 53] or physical activity [37, 127].

### 3.3 Risk Factors for Childhood Obesity During Pregnancy

We identified 135 publications from observational studies and two publications from RCTs that assessed 46 different risk factors during pregnancy (Figure 3A_2_,B_2_, Table S3). Most frequently studied characteristics were parental lifestyle factors and birth characteristics. Most risk factors were assessed in mother and child. Only six publications reported paternal data [34, 47, 75, 83, 188, 190].

Twenty‐nine publications from observational studies focused on parental physical factors. The majority investigated the association of (excessive) maternal gestational weight gain with childhood obesity. Fourteen [30, 39, 59, 74, 82, 99, 101, 130, 131, 132, 148, 153, 184, 189] of 16 publications based on observational studies reported that higher gestational weight gain was associated with an increased risk of childhood obesity. Twenty‐five [29, 34, 41, 42, 50, 67, 68, 78, 86, 99, 120, 125, 129, 134, 143, 145, 146, 148, 149, 153, 155, 157, 165, 179, 181] out of 31 publications from observational studies showed that maternal smoking during pregnancy was associated with a higher risk of childhood obesity. Ten publications from observational studies [31, 40, 46, 66, 77, 91, 95, 120, 125, 165] and one RCT [27] on maternal diet during pregnancy reached no consensus on the association with childhood obesity, partly because of the diversity of diets investigated. No consistent evidence was found for maternal age, socioeconomic status, ethnicity, parity, employment, relationship status, psychological stress or disorders, maternal or paternal education, and paternal age as risk factors for childhood obesity. Lower household income was reported within three [149, 187, 190] out of four publications from observational studies as a risk factor for obesity in childhood.

Forty‐nine publications focused on pregnancy complications as risk factors for childhood obesity, mainly gestational diabetes and mode of delivery. Gestational diabetes was identified as a risk factor for childhood obesity in 12 [30, 49, 65, 68, 71, 76, 107, 108, 126, 133, 140, 141] out of 21 publications based on observational studies. Within 14 [28, 30, 35, 56, 68, 80, 97, 124, 139, 148, 149, 159, 161, 194] out of 22 studied populations, caesarean section was identified as a risk factor for childhood obesity. Seven publications based on observational studies [48, 56, 78, 114, 144, 153, 197] and one RCT [147] reported no association between caesarean section and childhood obesity.

The most commonly reported risk factor for childhood obesity was birthweight, assessed continuously. Twenty [24, 46, 47, 54, 94, 99, 109, 110, 129, 137, 145, 154, 157, 162, 173, 177, 178, 179, 184, 197] of 28 publications from observational studies showed that a higher birthweight was associated with a higher risk of childhood obesity. Seventeen [33, 36, 61, 69, 83, 88, 92, 93, 98, 106, 117, 133, 135, 148, 149, 152, 171] out of 18 publications based on observational studies identified large for gestational age (LGA) as a risk factor for childhood obesity. For small for gestational age (SGA) at birth, findings were less consistent. Four publications from observational studies [32, 52, 154, 195] identified SGA as a risk for childhood obesity; however, eight publications based on observational studies [33, 44, 78, 96, 101, 133, 149, 152] reported that SGA was associated with a lower risk of childhood obesity. Most publications based on observational studies assessing gestational age at birth [30, 70, 116, 129, 178] or preterm and late‐term birth [21, 99, 121, 153] reported no evidence for a higher risk of childhood obesity.

Only three publications [40, 160, 164] explored different cord blood biomarkers in relation to childhood obesity. Risk factors were fatty acids [40], leptin and adiponectin [160], and differences in metabolomics [164], and no consensus on associations with childhood obesity was reached.

### 3.4 Risk Factors for Childhood Obesity During Infancy

We identified 76 publications from observational studies and three studies based on RCTs that assessed seven different exposures during infancy (Figure 3A_3_, B_3_). The most frequently studied characteristic was the feeding pattern. All risk factors were assessed in the child. Twenty [33, 43, 68, 72, 78, 82, 90, 101, 118, 123, 136, 138, 153, 156, 157, 171, 176, 179, 182, 193] out of 30 publications based on observational studies on breastfeeding reported that a breastfed child had a lower risk of obesity as compared to a non‐breastfed child. Furthermore, 15 publications based on observational studies [26, 41, 60, 72, 81, 99, 115, 116, 122, 146, 166, 172, 176, 180, 190] reported that longer breastfeeding duration was associated with a lower risk of childhood obesity; however, two publications based on one RCT [112, 113] and 13 publications based on observational studies [46, 50, 58, 70, 87, 89, 119, 128, 138, 158, 162, 178, 182] did not report an effect of the duration of breastfeeding on childhood obesity. All 16 publications based on observational studies [22, 23, 44, 57, 93, 96, 101, 104, 121, 129, 148, 149, 150, 163, 168, 178] on body mass index (BMI) or weight gain during infancy reported that higher infant BMI or weight gain was associated with a higher risk of childhood obesity.

### 3.5 Risk of Bias Assessment for Quality and Synthesis

**FIGURE 4:**
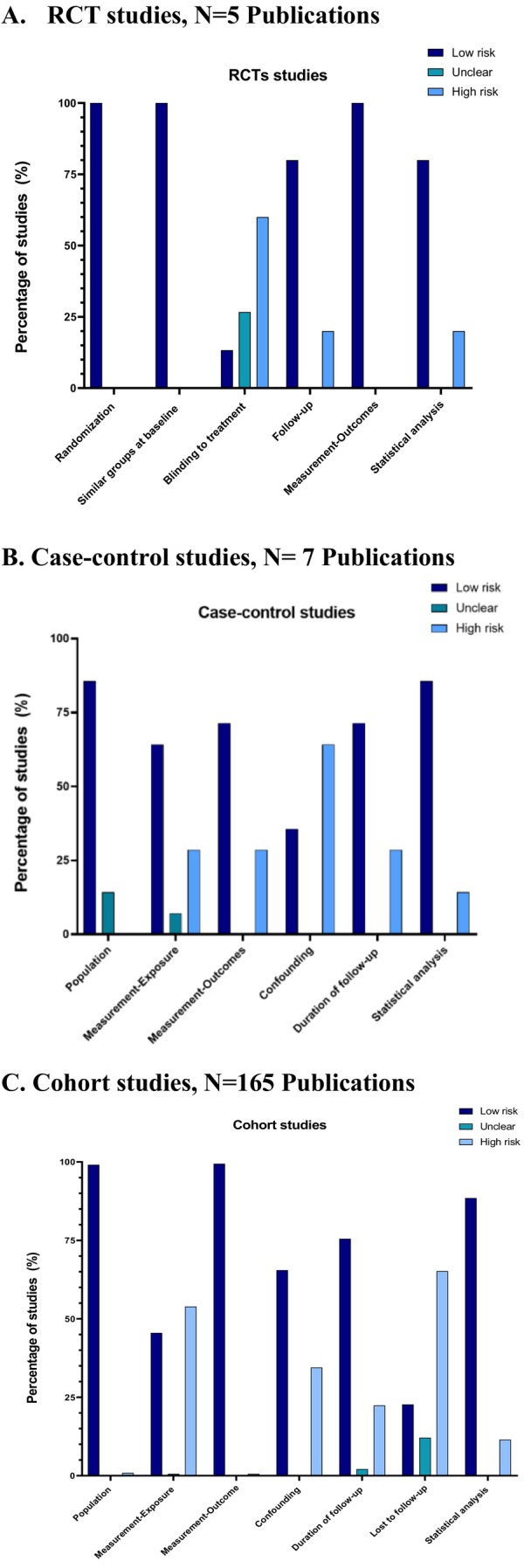
Risk for bias in different domains*. Numbers and domains based on JBI criteria and bias assessment; see Table S1 and Figures S1A–C–S3A–D for details. (A) RCT studies, *N* = 5 Publications. (B) Case–control studies, *N* = 7 Publications. (C) Cohort studies, *N* = 165 Publications.

Level of evidence was generally scored as moderate quality, mainly due to suboptimal assessment of the exposure or outcome, inadequate blinding of participants and researchers in RCTs, high risk of residual confounding, short duration of follow‐up, and high loss to follow‐up (Figure 4). Six percent of included studies were of low quality, consisting of one RCT and 10 cohort studies. Figures S1A–C–S3A–D show the detailed bias assessments for all included studies based on the JBI checklist.

### 3.6 Quality Assessment of Risk Factors for Prediction and Prevention

In total, we identified 23 risk factors consistently associated with childhood obesity, which included: maternal prepregnancy/pregnancy weight/overweight/obesity (hereafter maternal prepregnancy and pregnancy weight status), prepregnancy/pregnancy smoking, caesarean section, gestational diabetes, antibiotic use, education, household income, sleep duration, gestational weight gain/excessive gestational weight gain (hereafter maternal gestational weight gain), environmental chemical exposure, paternal pregnancy weight/overweight/obesity, continuous fetal growth, birthweight/high birthweight/LGA, breastfeeding/duration of breastfeeding, milk formula content, infant BMI/weight/and BMI/weight gain.

We evaluated the strength of these risk factors based on their potential for prediction and prevention strategies (Figure S4). In preconception, only maternal weight status scored strong or very strong on methodological aspects, reflection of the study objective, predictability, and modifiability. In pregnancy, maternal smoking, weight status, gestational weight gain, and birthweight/LGA scored strong or higher on methodological aspects, reflection of the study objective, prediction, and modifiability. In infancy, only breastfeeding (yes) and infant BMI/weight change scored strong or very strong for their methodological aspects, reflection of the study objective, potential as predictors, and to be modifiable on an individual level.

## 4 Discussion

In this systematic review, we identified 172 publications from observational and five publications from intervention studies from 37 countries that studied risk factors for childhood obesity in the first 1000 days of life. Most studies were performed in high‐income countries and focused on the identification of risk factors in pregnancy. Twenty‐three risk factors were consistently associated with the risk of childhood obesity. Higher maternal prepregnancy weight, higher gestational weight gain, maternal smoking during pregnancy, higher birthweight, large size for gestational age at birth, no breastfeeding, and higher infant weight gain were the strongest risk factors for childhood obesity and need to be considered in future prediction or prevention strategies. The overall level of evidence was generally moderate due to unreliable measurements of exposures, short follow‐up duration and loss to follow‐up, and the risk of residual confounding.

This review provides the most comprehensive, systematic, up‐to‐date information on family‐based risk factors for childhood obesity from preconception until infancy, thereby covering the full first 1000 days of life. Due to the large heterogeneity between studies, we could not perform meta‐analyses. Despite increasing awareness of the importance of the preconception period for offspring health, only a remarkably low number of studies focused on risk factors in this potential critical period. Maternal prepregnancy weight status was consistently associated with childhood obesity, but no consistent effects were found for other maternal lifestyle factors. The transgenerational effects of increased maternal prepregnancy weight on childhood obesity risk pose a major public health concern, as obesity often progresses into adulthood, subsequently causing increased risks for the next generation [198]. Only one publication focused on paternal factors and reported that higher paternal prepregnancy BMI was associated with a higher childhood obesity risk [145]. Adverse paternal sociodemographic and lifestyle factors have already been shown to be associated with adverse pregnancy outcomes [199]. Animal studies showed that multiple paternal factors from preconception onwards are associated with an increased risk of offspring obesity, with the strongest effects for paternal obesity, hyperglycemia, and smoking [200]. The very limited number of studies that focused on the preconception period may be related to major research challenges. Couples actively planning pregnancy are a very difficult population to target, often not yet in contact with health care professionals. Across the world, there is a high diversity in awareness and coverage regarding preconception care, and in those countries with preconception care, attendance is generally low [201]. Not all couples included in a preconception cohort will develop the outcome of interest, and clearly long‐term follow‐up is needed, which results in the need for a large study population from inclusion onwards, high risks of attrition, and high costs. However, worldwide there are now successful examples of preconception cohort studies, such as the population‐based Generation R *Next* Study [202], the tertiary hospital‐based Rotterdam Periconceptional Cohort (Predict Study) [203], and the population‐based Singapore Preconception Study of Long‐term Maternal and Child Outcomes (S‐PRESTO) [204], allowing future novel longitudinal studies on childhood obesity development from preconception onwards. Strong international collaboration is needed to share knowledge on the set‐up and management of preconception cohorts, to enable harmonized data collection, and to collaborate in future large‐scale meta‐analyses to identify novel and consistent preconception risk factors for childhood obesity development.

As expected, pregnancy emerged as the most studied period for childhood obesity risk, with most studies focusing on maternal and fetal risk factors. Suboptimal maternal gestational weight status and gestational weight gain were consistently associated with the risk of childhood obesity. Gestational diabetes and gestational hypertensive disorders tended to be associated with a higher risk of childhood obesity, but this was not consistent in moderate to high‐quality studies. Indeed, a previous individual‐participant‐data meta‐analysis among 160,757 mother‐offspring pairs from European and North American birth cohorts also reported that no effect of gestational diabetes or gestational hypertensive disorders was present on childhood obesity after adjustment for maternal prepregnancy BMI [205]. A higher birthweight and LGA were strong risk factors for childhood obesity, whereas associations for SGA and preterm birth were inconclusive. Children born SGA may be constitutionally small or have suffered from intra‐uterine fetal growth restriction. Differences in this pathophysiology may partly explain these inconsistent findings [206]. A lower household income and lower maternal and paternal education levels tended to be associated with a higher risk of childhood obesity, but only inconsistently in less than five studies of at least moderate quality. As these findings are less consistent, generalizability is more difficult. Possibly, these effects are more country‐specific and influenced by community and social support practices per region. Several moderate and high‐quality studies reported an association between cesarean section and a higher risk for childhood obesity. All were publications based on observational studies, and the only included RCT did not support that association, suggesting that residual confounding may play a role in these observed associations. Maternal antibiotics during pregnancy were associated with childhood obesity in 50% of studies, but the number of studies was low, and associations were only found in females [151] or not in all investigated age groups [111, 183], so no generalized conclusion can be drawn. No consistent associations were present for maternal or paternal age, ethnicity, or parity.

In infancy, infant weight gain and feeding patterns were mostly studied, with the strongest associations present for higher infant weight or BMI gain with higher risks of obesity. Being breast‐fed and a longer duration of breastfeeding were associated with lower risks of childhood obesity within the majority of reported observational studies. The only two publications based on the same RCT [112, 113] did not identify a significant association between the duration of breastfeeding and childhood obesity. It is possible that, because of the substantial overlap in breastfeeding duration and exclusivity in the intervention and control groups, the PROBIT trial intervention did not produce a sufficiently large difference between the intervention and control groups to detect differences in childhood obesity risk, caused by increased duration and exclusivity of breastfeeding [112]. On the other hand, residual confounding may be an important issue in observational studies, as the observed beneficial effects of breastfeeding might be explained by the characteristics of the families who choose to breastfeed, instead of the breastfeeding itself [207]. It is remarkable that we identified no studies on the role of different components of breastmilk and only three studies on differences in infant formula composition [90, 115, 186].

Our systematic review showed that many studies investigated similar, well‐established maternal and birth‐related risk factors for childhood obesity. We partly identified similar risk factors for childhood obesity as two systematic reviews performed over a decade ago [11, 12]. Due to the large heterogeneity between studies, including the large variety of measured exposures and their definitions, different study populations and study settings, different ages and diagnostic cut‐offs in outcome assessment, and large differences in statistical approaches and adjustment for confounders, we could not perform meta‐analyses [208]. To gain the most accurate and complete overview of the existing literature on risk factors for childhood obesity, we included all potential definitions of childhood obesity, as defined by individual studies. Even though different reference charts to define childhood obesity with assessments at different ages are useful for country‐specific research questions, our findings also clearly highlight the urgent need for datasets with harmonized and standardized data collection for both exposures and outcomes in childhood obesity‐related observational and intervention research. This will enable large‐scale collaborations and meta‐analyses, crucial for identification of novel risk factors, studies on interactive effects between risk factors, subgroup analyses, and the development of accurate, generalizable prediction models for childhood obesity. A very limited number of studies focused on paternal physical, lifestyle, and physiological factors from preconception onwards, family‐based environmental factors, or novel risk factors in infancy, such as responsive feeding, screen time, or neurodevelopmental factors. Remarkably, we also identified a major knowledge gap with regard to research on novel non‐invasive biomarkers for childhood obesity, even though omics techniques have developed rapidly in the past decade. The majority of studies on novel biomarkers did not meet our inclusion criteria, mainly due to cross‐sectional study designs, non‐human study populations, and very short follow‐up with childhood obesity outcomes assessed before the age of 2 years. Clearly, there is large potential yet to be capitalized. Evidence is increasing on the role of environmental exposures in human health, and investigation of their role in the development of childhood obesity is warranted, as they are ubiquitous and represent a unique opportunity for governmental and political intervention on a population level. Additionally, omics data, including metabolomics, epigenomics, and genomics, may have the potential to improve early‐life risk prediction of childhood obesity, especially when combined with advanced AI‐based modeling approaches. For example, one recent small study among 30 children aged 1 year observed stable DNA methylation patterns in saliva from children that were associated with adverse growth patterns in the first year of life, but effects on childhood obesity development remain to be studied [209]. Further collaborative studies, moving beyond the standard risk factors for childhood obesity, are urgently needed to identify detailed patterns of non‐invasive biomarkers associated with childhood obesity in human populations.

Quality assessment of the included publications showed an overall moderate quality, partly because of the methods used for exposure and/or outcome measurements, high risk for residual confounding, short duration of follow‐up, and high loss to follow‐up. Especially for exposure measurements, self‐reported questionnaires were widely used. They are easy to use for both participants and researchers, accessible, and low in cost compared to, for example, research visits to a dedicated research centre, but also more prone to measurement error. Importantly, the majority of the publications (80%) measured the outcome of childhood obesity via measurements at research centres or medical records. Ninety‐seven percent of publications were based on observational cohort or case–control studies. The major limitation of these observational studies is confounding. Various family‐based socio‐demographic, nutritional, lifestyle‐related, and genetic characteristics may explain specific observed associations of early‐life risk factors with childhood obesity development. In approximately 40% of publications, adjustment for confounders was inadequate, and even in publications with adequate adjustment, residual confounding may be present. Randomized controlled trials are considered as the gold standard for causality studies but are difficult to perform for many of the early‐life risk factors for childhood obesity development. Randomized controlled trials focused on influencing determinants of early‐life risk factors, such as stimulating breastfeeding in the PROBIT trial [112, 113] or optimizing maternal diet and exercise to reduce excessive gestational weight gain [210], are strategies to better study the impact and causality of these early‐life exposures on childhood obesity development. More sophisticated study designs in observational studies also offer a way forward to obtain further insight into the role of confounding in the observed associations, including sibling comparison studies, maternal and paternal offspring comparison analyses, and Mendelian randomization studies, but few have been performed so far [211]. One large sibling comparison study examined the associations between breastfeeding initiation, caesarean delivery, prenatal smoking, and gestational diabetes with childhood obesity at ages 2 and 5 years and concluded that unmeasured genetic, environmental, and familial factors are likely confounding the observed associations [68]. Conversely, a maternal–paternal–offspring comparison analysis showed that both higher maternal and paternal prepregnancy BMI were associated with childhood overweight and obesity at 6 years, with stronger associations present for maternal prepregnancy BMI, suggesting that maternal prepregnancy BMI may at least partly influence offspring obesity risk through direct intrauterine mechanisms [212]. Thus, improvements in future research can mainly be established by investments in the exposure assessments, retention strategies, approaches to minimize residual confounding with detailed data collection on covariates, and use of sophisticated observational study designs or by conducting well‐designed intervention studies targeting risk factors from preconception onwards with long‐term offspring follow‐up.

Despite the limitations of the identified evidence, our findings are important from an etiological perspective and provide novel insights to improve prediction and prevention strategies from the start of life onwards. Animal studies strongly suggest that maternal prepregnancy overweight and obesity, high gestational weight gain, and smoking during pregnancy may change the intra‐uterine environment, affecting embryonic and fetal structural and functional development, fat deposition, and the development of the hypothalamic‐endocrine system that controls appetite and energy metabolism, predisposing children to a higher risk of obesity in later life [198, 211, 213, 214]. High birth weight and childhood obesity have been linked to substantial genetic pleiotropic effects, suggesting a shared genetic basis in the regulation of birth weight and childhood obesity [215]. Infancy reflects the greatest proportional weight gain in postnatal life and might be a critical period for the development of energy balance mechanisms. Early exposure to excess and dysfunctional fat mass might trigger the development of metabolic changes in childhood [216]. These developmental programming mechanisms may act in addition to the shared family‐based environment, with potential interactions between early‐life risk factors that together may contribute to the development of childhood obesity [198].

The first 1000 days of life offer a unique opportunity for prevention, because of parental motivation to make lifestyle changes to benefit the health of their unborn child and because parents‐to‐be are in contact with the health care system [16]. Improved risk selection from the start of life onwards is critical to identify those families who will benefit most from childhood obesity prevention strategies. For risk selection, causality is not an issue as variables need to have a strong predictive value regardless of whether a causal relationship exists [20]. We identified several risk factors, which are even already partly obtained in prenatal care, that may also aid prediction strategies for childhood obesity from the start of life onwards, such as maternal prepregnancy obesity, maternal smoking, and birth weight. In the past decades, multiple prediction models to assess the risk of childhood overweight/obesity have been developed, but the majority of these models included risk factors obtained during childhood and did not specifically focus on the first 1000 days of life, with especially the preconception period not being adequately used [217]. To model the complexity of joint effects and the interaction of multiple risk factors related to childhood obesity, more use of complex methods such as machine learning and artificial neural networks is needed. Subsequent prevention strategies focused on improving the identified early‐life risk factors from a clinical and population perspective are needed, targeting those families at higher risk of offspring obesity, preferably integrated with implementation studies to evaluate risk selection, intervention effects, multi‐risk factor intervention approaches versus single risk factor intervention approaches, and to optimize intervention delivery methods.

## 5 Conclusion

We showed that 23 risk factors in early life are consistently associated with a higher risk of childhood obesity. Higher maternal prepregnancy weight and gestational weight gain, maternal smoking during pregnancy, higher birthweight and large‐size‐for‐gestational‐age‐at‐birth, no breastfeeding, and higher infant weight gain are most strongly related to childhood obesity risk. These findings are relevant for early life risk prediction and insight into the strongest potential modifiable factors from a clinical, governmental, and industrial perspective.

## Author Contributions

S.M.B. drafted the initial version of the manuscript and completed the review, extraction, and quality assessment of papers. A.S.J.K. drafted the initial version of the manuscript and completed the review, extraction, and quality assessment of papers. F.J.R.‐O. completed the review, extraction, and quality assessment of papers and provided critical intellectual feedback. M.B.‐H. provided extraction of the data, provided critical intellectual feedback, and completed quality assessment of papers. E.F.‐V. provided extraction of the data and provided critical intellectual feedback. M.A.B. provided extraction of the data and provided critical intellectual feedback. M.C.C. provided extraction of the data and provided critical intellectual feedback. J.v.D. set up the initial aim of the study and study protocol, provided extraction of the data, and provided critical intellectual feedback. P.I. provided extraction of the data and provided critical intellectual feedback. K.K. provided extraction of the data and provided critical intellectual feedback. C.A.v.L.‐B. provided extraction of the data and provided critical intellectual feedback. Á.G. completed the review, extraction, and quality assessment of papers and provided critical intellectual feedback. R.G. set up the initial aim of the study and study protocol, completed the review, extraction, and quality assessment of papers, drafted the initial version of the manuscript, and led this expert group of the European branch of the International Life Sciences Institute, ILSI Europe, as chair. All authors approved the final version for publication.

## Conflicts of Interest

The authors declare no conflicts of interest.

## Supporting information


**Text S1:** Search terms for the systematic review.
**Table S1:** Handling of the JBI criteria.
**Table S2:** Criteria template for quality assessment of the risk factors.
**Table S3:** Associations of each early ‐life risk factor with childhood obesity per critical period.
**Figure S1:**–3. Bias assessment of the included studies.
**Figure S4:** Quality assessment of the 23 consistently associated risk factors with childhood obesity*.

## Data Availability

The data that support the findings of this study are available from the corresponding author upon reasonable request.
