# Supplementary material for: Risk Factors in the First 1000 Days of Life Associated With Childhood Obesity: A Systematic Review and Risk Factor Quality Assessment
Source: Obes Rev. 2025 Nov 19;27(2):e70025. doi: 10.1111/obr.70025 (PMC12812507; doi:10.1111/obr.70025)
Supplement: Supplementary file 1 — Text S1: Search terms for the systematic review. Table S1: Handling of the JBI criteria. Table S2: Criteria template for quality assessment of the risk factors. Table S3: Associations of each early ‐life risk factor with childhood obesity per critical period. Figure S1:–3. Bias assessment of the included studies. Figure S4: Quality assessment of the 23 consistently associated risk factors with childhood obesity*. [file OBR-27-e70025-s001.pdf]

Supplemental material – Supporting Information

Risk factors in the first 1000 days of life associated with childhood obesity: a systematic review and risk factor quality assessment.

Sophia M Blaauwendraad MD[1,2]*, Arwen SJ Kamphuis MD[1,2]*, Francisco Javier Ruiz-Ojeda PhD[3,4,5,6,7]*, Marco Brandimonte-Hernández MSc[3], Eduard Flores-Ventura MSc[8], Marieke Abrahamse-Berkeveld PhD[9], Maria Carmen Collado PhD[8], Janna A van Diepen PhD[10], Patricia Iozzo MD PhD[11], Karen Knipping MSc[12], Carolien A van Loo-Bouwman PhD[13], Ángel Gil PhD[3,4,5,8#], Romy Gaillard MD, PhD[1,2#]

*These authors contributed equally
#These authors contributed equally

[1]The Generation R Study Group, Erasmus MC, University Medical Center, Rotterdam, the Netherlands
[2]Department of Pediatrics, Erasmus MC, University Medical Center, Rotterdam, the Netherlands
[3]Department of Biochemistry and Molecular Biology II, School of Pharmacy, University of Granada, 18071, Granada, Spain
[4]Institute of Nutrition and Food Technology "José Mataix", Centre of Biomedical Research, University of Granada, Avda. del Conocimiento s/n. 18016 Armilla, Granada, Spain
[5]Instituto de Investigación Biosanitaria IBS.GRANADA, Complejo Hospitalario Universitario de Granada, Granada 18014, Spain
[6]Adipocytes and Metabolism Unit. Helmholtz Diabetes Center, Helmholtz Munich, Munich, Germany
[7] CIBEROBN (CIBER Physiopathology of Obesity and Nutrition), Instituto de Salud Carlos III, 28029 Madrid, Spain
[8]Institute of Agrochemistry and Food Technology-National Research Council (IATA-CSIC), Augustin Escardino 7, 46980, Paterna, Valencia, Spain
[9] Danone Nutricia Research, 3584 CT Utrecht, The Netherlands
[10] Reckitt Benckiser/Mead Johnson Nutrition, 6545 CJ Nijmegen, The Netherlands
[11] Institute of Clinical Physiology, National Research Council (CNR), Via G. Moruzzi 1, 56124, Pisa, Italy
[12] Ausnutria B.V., 8025 BM Zwolle, The Netherlands
[13] Yili Innovation Center Europe, 6708 WH Wageningen, The Netherlands

Corresponding author: Romy Gaillard, The Generation R Study Group, Erasmus University Medical Center, PO Box 2040, 3000 CA Rotterdam, the Netherlands. E-mail: r.gaillard@erasmusmc.nl; publications@ilsieurope.be

Supporting information

Text S1. Search terms for the systematic review.

Table S1. Handling of JBI criteria

Table S2. Guideline on quality assessment of risk factors.

Table S3. Associations of each early-life risk factor with childhood obesity per critical period..

Figure S1-3. Bias assessment of the included studies.

Figure S4. Quality assessment of the 23 consistently associated risk factors with childhood obesity.

Text S1. Search terms for the systematic review.

EMBASE
https://www.embase.com/#advancedSearch/default – 10659 records
('pregnancy'/exp OR 'pregnancy' OR 'pregnant woman'/exp OR 'pregnant woman' OR 'mother'/exp OR 'mother' OR 'prenatal exposure'/exp OR 'prenatal exposure' OR 'pregnancy outcome'/exp OR 'pregnancy outcome' OR 'pregnancy disorder'/exp OR 'pregnancy disorder' OR 'pregnancy complication'/exp OR 'pregnancy complication' OR 'high-risk pregnancy'/exp OR ‘adolescent pregnancy’/exp OR ‘maternal smoking’/exp OR 'prenatal period'/exp OR 'prenatal period' OR 'prenatal growth'/exp OR 'prenatal growth' OR 'fetus disease'/exp OR 'fetus disease' OR 'newborn disease'/exp OR 'newborn disease' OR 'low birth weight'/exp OR 'low birth weight' OR 'umbilical cord blood'/exp OR 'umbilical cord blood' OR 'infant disease'/exp OR 'infant disease' OR 'adolescent pregnancy'/exp OR 'adolescent pregnancy' OR gravidit*:ab,ti,kw OR gestation*:ab,ti,kw OR pregnan*:ab,ti,kw OR mother*:ab,ti,kw OR prenatal*:ab,ti,kw OR preeclamp*:ab,ti,kw OR 'pre eclamp*':ab,ti,kw OR (((prematur* OR preterm* OR live*) NEAR/3 (labor OR birth OR labour OR delivery OR ruptur*)):ab,ti,kw) OR birthweight*:ab,ti,kw OR (((birth OR neonat* OR newborn OR infant*) NEAR/3 weight*):ab,ti,kw) OR (((infant* OR child*) NEAR/3 ('breast feed*' OR breastfeed* OR 'milk composit*' OR weaning)):ab,ti,kw) OR ((infant* NEAR/3 (biomarker* OR saliva OR fecal OR urine OR hair)):ab,ti,kw) OR postpartum:ab,ti,kw OR 'post partum':ab,ti,kw OR puerperium:ab,ti,kw OR (((fetal OR foetus OR fetus OR newborn OR neonat* OR infant* OR intrauterin* OR 'intra uterin*') NEAR/3 (diseas* OR abnormal* OR growth* OR infect*)):ab,ti,kw) OR ((gestational NEAR/3 (diabet* OR hypertens* OR weight*)):ab,ti,kw) OR ((placenta* NEAR/3 abruption):ab,ti,kw) OR ((('small for date' OR 'small for gestational age' OR 'large for date' OR 'large for gestational age') NEAR/3 infant*):ab,ti,kw) OR preconcept*:ab,ti,kw OR maternal*:ab,ti,kw OR paternal*:ab,ti,kw OR father*:ab,ti,kw OR parent*:ab,ti,kw OR 'cord blood*':ab,ti,kw OR 'fetal blood*':ab,ti,kw OR lifestyle*:ab,ti,kw OR sociodemographic*:ab,ti,kw) **AND** ('follow up'/exp OR 'follow up' OR 'longitudinal study'/exp OR 'longitudinal study' OR 'prospective study'/exp OR 'prospective study' OR 'retrospective study'/exp OR 'retrospective study' OR 'cohort analysis'/exp OR 'cohort analysis' OR 'prediction'/exp OR 'prediction' OR followup*:ab,ti,kw OR 'follow up*':ab,ti,kw OR longitudinal*:ab,ti,kw OR prospective*:ab,ti,kw OR retrospective*:ab,ti,kw OR cohort*:ab,ti,kw OR predict*:ab,ti,kw) **AND** ((((obes* OR hypertension* OR diabetes* OR diabetic* OR bmi OR 'body mass*' OR 'waist circum*' OR 'fat distribut*' OR dyslipidemia* OR lipidemia OR lipoedema OR 'glucos* intoleranc*' OR 'glucos* toleranc*' OR overweight* OR overnutrition*) NEAR/6 (child* OR progeny OR offspring*)):ab,ti,kw) OR (((metabolic* OR cardiometabolic* OR 'insulin resist*' OR dysmetabolic* OR metabolism*) NEAR/2 (syndrom* OR diseas* OR outcome* OR disorder*) NEAR/9 (child* OR progeny OR offspring*)):ab,ti,kw) OR (((increas* OR elevat* OR high*) NEAR/2 (bloodpress* OR 'blood press*') NEAR/9 (child* OR progeny OR offspring*)):ab,ti,kw)) **NOT** (('animal'/exp OR animal OR animal*:de OR 'nonhuman'/exp OR nonhuman) NOT ('human'/exp OR 'human')) AND [english]/lim NOT ([conference abstract]/lim OR [preprint]/lim) NOT ('case report'/exp OR 'case report' OR 'case report*':ti)

Medline
https://ovidsp.dc1.ovid.com/ovid-b/ovidweb.cgi – 11364 results.
(exp Pregnancy/ OR Pregnant Women/ OR exp Parents/ OR exp Pregnancy Complications/ OR Pregnancy Outcome/ OR exp Infant, Newborn, Diseases/ OR exp Infant, Low Birth Weight/ OR Fetal Blood/ OR exp Pregnancy, High-Risk/ OR exp Pregnancy in Adolescence/ OR exp Biomarkers/ OR exp Cardiometablic Risk Factors/ OR exp sociodemographic factors/ OR exp maternal exposure/ OR exp Feeding Behavior/ OR exp Infant, Newborn, Disease/ OR (gravidit* OR gestation* OR pregnan* OR prenatal* OR maternal* OR preeclamp* OR pre-eclamp* OR ((prematur* OR preterm* OR live*) ADJ3 (labor OR birth OR labour OR delivery OR ruptur*)) OR birthweight* OR ((birth OR neonat* OR newborn OR infant*) ADJ3 (weight*)) OR ((infant* OR child*) ADJ3 (breast-feed* OR breastfeed* OR milk-composit* OR weaning)) OR ((infant*) ADJ3 (biomarker* OR saliva OR fecal OR urine OR hair)) OR postpartum OR post-partum OR puerperium OR ((fetal OR foetus OR fetus OR newborn OR neonat* OR infant* OR intrauterin* OR intra-uterin*) ADJ3 (diseas* OR abnormal* OR growth* OR infect*)) OR ((gestational) ADJ3 (diabet* OR hypertens* OR weight*)) OR ((placenta*) ADJ3 (abruption)) OR ((small-for-date OR small-for-gestational-age OR large-for-date OR large-for-gestational-age) ADJ3 (infant*)) OR preconcept* OR maternal* OR paternal* OR father* OR parent* OR cord-blood* OR fetal-blood* OR lifestyle* OR sociodemographic*).ab,ti,kf.) **AND** (exp Cohort Studies/ OR (followup* OR follow-up* OR longitudinal* OR prospective* OR retrospective* OR cohort* OR predict*).ab,ti,kf.) **AND** (exp Pediatric Obesity/ OR (((obes* OR hypertension* OR diabetes* OR diabetic* OR BMI OR body-

mass* OR waist-circum* OR fat-distribut* OR dyslipidemia* OR lipidemia OR lipoedema OR glucos*-intoleranc* OR glucos*-toleranc* OR overweight* OR overnutrition*) ADJ6 (child* OR progeny OR offspring*)) OR ((metabolic* OR cardiometabolic* OR insulin-resist* OR dysmetabolic* OR metabolism*) ADJ2 (syndrom* OR diseas* OR outcome* OR disorder*) ADJ9 (child* OR progeny OR offspring*)) OR ((increas* OR elevat* OR high*) ADJ2 (bloodpress* OR blood-press*) ADJ9 (child* OR progeny OR offspring*))).ab,ti,kf.) NOT (exp animals/ NOT humans/) AND (english).lg NOT (news OR congres* OR abstract* OR book* OR chapter* OR dissertation abstract*).pt. NOT (Case Reports/ OR case-report*.ti.)

Web of Science

https://www.webofscience.com/wos/woscc/advanced-search - 10034 records

TS=(((gravidit* OR gestation* OR pregnan* OR mother* OR prenatal* OR preeclamp* OR pre-eclamp* OR ((prematur* OR preterm* OR live*) NEAR/2 (labor OR birth OR labour OR delivery OR ruptur*)) OR birthweight* OR ((birth OR neonat* OR newborn OR infant*) NEAR/2 (weight*)) OR ((infant* OR child*) NEAR/2 (breast-feed* OR breastfeed* OR milk-composit* OR weaning)) OR ((infant*) NEAR/2 (biomarker* OR saliva OR fecal OR urine OR hair)) OR postpartum OR post-partum OR puerperium OR ((fetal OR foetus OR fetus OR newborn OR neonat* OR infant* OR intrauterin* OR intra-uterin*) NEAR/2 (diseas* OR abnormal* OR growth* OR infect*)) OR ((gestational) NEAR/2 (diabet* OR hypertens* OR weight*)) OR ((placenta*) NEAR/2 (abruption)) OR ((small-for-date OR small-for-gestational-age OR large-for-date OR large-for-gestational-age) NEAR/2 (infant*)) OR preconcept* OR maternal* OR paternal* OR father* OR parent* OR cord-blood* OR fetal-blood* OR lifestyle* OR sociodemographic*)) AND ((followup* OR follow-up* OR longitudinal* OR prospective* OR retrospective* OR cohort* OR predict*)) AND ((((obes* OR hypertension* OR diabetes* OR diabetic* OR BMI OR body-mass* OR waist-circum* OR fat-distribut* OR dyslipidemia* OR lipidemia OR lipoedema OR glucos*-intoleranc* OR glucos*-toleranc* OR overweight* OR overnutrition*) NEAR/5 (child* OR progeny OR offspring*)) OR ((metabolic* OR cardiometabolic* OR insulin-resist* OR dysmetabolic* OR metabolism*) NEAR/2 (syndrom* OR diseas* OR outcome* OR disorder*) NEAR/9 (child* OR progeny OR offspring*)) OR ((increas* OR elevat* OR high*) NEAR/2 (bloodpress* OR blood-press*) NEAR/9 (child* OR progeny OR offspring*)))) NOT ((animal* OR rat OR rats OR mouse OR mice OR murine OR dog OR dogs OR canine OR cat OR cats OR feline OR rabbit OR cow OR cows OR bovine OR rodent* OR sheep OR ovine OR pig OR swine OR porcine OR veterinar* OR chick* OR zebrafish* OR baboon* OR nonhuman* OR primate* OR cattle* OR goose OR geese OR duck OR macaque* OR avian* OR bird* OR fish*) NOT (human* OR patient* OR women OR woman OR men OR man))) AND LA=(English) AND DT=(Article OR Review OR Letter OR Early Access)

Scopus

Scopus - Advanced search – 1631 records

(TITLE-ABS-KEY ((gravidit* OR gestation* OR pregnan* OR mother* OR prenatal* OR preeclamp* OR pre-eclamp* OR ((prematur* OR preterm* OR live*) PRE/3 (labor OR birth OR labour OR delivery OR ruptur*)) OR birthweight* OR ((birth OR neonat* OR newborn OR infant*) PRE/3 (weight)) OR ((infant* OR child*) PRE/3 (breast-feed* OR breastfeed* OR milk-composit* OR weaning)) OR ((infant*) PRE/3 (biomarker* OR saliva OR fecal OR urine OR hair)) OR postpartum OR post-partum OR puerperium OR ((fetal OR foetus OR fetus OR newborn OR neonat* OR infant* OR intrauterin* OR intra-uterin*) PRE/3 (diseas* OR abnormal* OR growth* OR infect*)) OR ((gestational) PRE/3 (diabet* OR hypertens* OR weight*)) OR ((placenta*) PRE/3 (abruption)) OR ((small-for-date OR small-for-gestational-age OR large-for-date OR large-for-gestational-age) PRE/3 (infant*)) OR preconcept* OR maternal* OR paternal* OR father* OR parent* OR cord-blood* OR fetal-blood* OR lifestyle* OR sociodemographic*))) AND (TITLE-ABS-KEY ("follow up" OR "longitudinal AND study" OR "prospective AND study" OR "retrospective AND study" OR "cohort AND analysis" OR "prediction" OR (followup* OR follow-up* OR longitudinal* OR prospective* OR retrospective* OR cohort* OR predict*))) AND (TITLE-ABS-KEY (((obes* OR hypertension* OR diabetes* OR diabetic* OR bmi OR body-mass* OR waist-circum* OR fat-distribut* OR dyslipidemia* OR lipidemia OR lipoedema OR glucos*-intoleranc* OR glucos*-toleranc* OR overweight* OR overnutrition*) PRE/6 (child* OR progeny OR offspring*)) OR (metabolic* OR cardiometabolic* OR insulin-resist* OR dymetabolic* OR metabolism*) PRE/2 (syndrom* OR diseas* OR outcome* OR disorder*) PRE/9 (child* OR progeny OR offspring*)) OR ((increas* OR elevat* OR high*) PRE/2 (bloodpress* OR blood-press*) PRE/9 (child* OR progeny OR offspring*))) AND

NOT ((INDEXTERMS (animals OR animal)) AND NOT (INDEXTERMS (humans OR human))) AND (LIMIT-TO (LANGUAGE,"English")) AND (EXCLUDE (DOCTYPE,"cp") OR EXCLUDE (DOCTYPE,"ch") OR EXCLUDE (DOCTYPE,"le"))

Cochrane
https://www.cochranelibrary.com/advanced-search/search-manager - 1896 records
((gravidit* OR gestation* OR pregnan* OR mother* OR prenatal* OR preeclamp* OR pre NEXT/1 eclamp* OR ((prematur* OR preterm* OR live*) NEAR/3 (labor OR birth OR labour OR delivery OR ruptur*)) OR birthweight* OR ((birth OR neonat* OR newborn OR infant*) NEAR/3 (weight*)) OR ((infant* OR child*) NEAR/3 (breast NEXT/1 feed* OR breastfeed* OR milk NEXT/1 composit* OR weaning)) OR ((infant*) NEAR/3 (biomarker* OR saliva OR fecal OR urine OR hair)) OR postpartum OR post NEXT/1 partum OR puerperium OR ((fetal OR foetus OR fetus OR newborn OR neonat* OR infant* OR intrauterin* OR intra NEXT/1 uterin*) NEAR/3 (diseas* OR abnormal* OR growth* OR infect*)) OR ((gestational) NEAR/3 (diabet* OR hypertens* OR weight*)) OR ((placenta*) NEAR/3 (abruption)) OR ((small NEXT/1 for NEXT/1 date OR small NEXT/1 for NEXT/1 gestational NEXT/1 age OR large NEXT/1 for NEXT/1 date OR large NEXT/1 for NEXT/1 gestational NEXT/1 age) NEAR/3 (infant*)) OR preconcept* OR maternal* OR paternal* OR father* OR parent* OR cord NEXT/1 blood* OR fetal NEXT/1 blood* OR lifestyle* OR sociodemographic*):ab,ti,kw) **AND** ((followup* OR follow NEXT/1 up* OR longitudinal* OR prospective* OR retrospective* OR cohort* OR predict*):ab,ti,kw) **AND** ((((obes* OR hypertension* OR diabetes* OR diabetic* OR BMI OR body NEXT/1 mass* OR waist NEXT/1 circum* OR fat NEXT/1 distribut* OR dyslipidemia* OR lipidemia OR lipoedema OR glucos* NEXT/1 intoleranc* OR glucos* NEXT/1 toleranc* OR overweight* OR overnutrition*) NEAR/6 (child* OR progeny OR offspring*)) OR ((metabolic* OR cardiometabolic* OR insulin NEXT/1 resist* OR dysmetabolic* OR metabolism*) NEAR/2 (syndrom* OR diseas* OR outcome* OR disorder*) NEAR/9 (child* OR progeny OR offspring*)) OR ((increas* OR elevat* OR high*) NEAR/2 (bloodpress* OR blood NEXT/1 press*) NEAR/9 (child* OR progeny OR offspring*))):ab,ti,kw)

Table S1. Handling of the JBI criteria.		
	**Reliable**	**Unreliable**
**Exposure measurements**	Obtained from medical records<br>Obtained from visits at research centres<br>Obtained from interviews	Self-reported<br>Self-reported questionnaires
**Outcome measurements**	Obtained from medical records<br>Obtained from visits at research centres<br>Obtained from interviews	Self-reported<br>Self-reported questionnaires
**Confounding**	Adjustment for at least:<br>- Socio-economic status/ income/educational level<br>- Maternal BMI<br>- Ethnicity<br>- Parity<br>- Maternal smoking during pregnancy<br>In case the investigated exposure is a birth-related characteristics: additional adjustment for:<br>- Gestational age or birth weight (depending on characteristic)	Not adjusted for (one or more) of the confounders stated in the reliable column
**Follow-up**	At least 5 years of follow-up to ensure enough time between the exposure and the outcome to develop*. From childhood onwards, there is a high risk for obesity to track into adulthood [13,14]	Less than 5 years
**Loss to follow-up**	Loss to follow-up < 20%<br>In case of more than 20% loss to follow up: statistical approaches as inverse probability weighting, censoring weights, non-response analysis	Loss to follow-up >20% and no statistical approaches used such as inverse probability weighting, censoring weights, non-response analysis
**Statistical approach**	Regression analyses (or other appropriate methods) adjusted for appropriate covariates	Qualitative/ correlation without any covariate adjustments.

Table S2. Criteria template for quality assessment of the risk factors.

	Methodological aspects (excluding study design)		Reflect/mark the study objective	Modifiable		Prediction
**Levels**	**Reproducibility, accuracy, standardization, stability (quality of the sample) and technical variation**	**Biological variation**	**A change in the marker is linked with a change in the endpoint in one or more target population(s)**	**Theoretically modifiable**	**Intervention studies**	
Very strong (+++)	Marker is highly reproducible (intra class correlation coefficient >0.9) and accurate (less than 1% deviation from 'correct' value), assay is highly standardized, sample is stable or can easily be made stable	Minimal variation and relevant effects highly superior to variation: effects likely to be observed between groups of tens of individuals	Generally, accepted marker (marker changes consistently linked with a change in the endpoint)	The risk factor is modifiable on an individual level and implementation of modifications in daily life is easy	A systematic review on intervention on this risk factor has been conducted and shows a positive effect of modification on the risk factor and outcome	>80% of moderate or high quality papers report an association in the same direction and at least 5 publications
Strong (++)	Marker is reproducible and accurate enough to detect biological meaningful changes, assay is standardized, sample is stable or can be made stable	High variation explainable (for example, circadian cycle, age, sex, BMI, ethnicity and genotype) and possible to correct it and relevant effects reproducibly superior to variation: effects likely to be observed between groups of fifties to hundreds of individuals	Described as a cause- and effect relationship, but not (yet) generally accepted as a marker, due to a lack of (specific) studies	The risk factor is modifiable on an individual level and implementation of modifications in daily life is complicated	Multiple intervention studies have been conducted showing a potential positive effect of modification on the risk factor and outcome	>65% of moderate or high quality papers report an association in the same direction and at least 5 publications
Medium (+)	Marker is reproducible and accurate enough for specific applications, assay is somewhat standardized and needs to be extensively processed or analysed fast	High variation explainable (for example, circadian cycle, age, sex, BMI, ethnicity and genotype) and possible to correct it and relevant effects reproducibly close to variation: effects may be observed between groups of fifties to hundreds of individuals	Body of evidence suggesting correlation, but cause and effect not established	Modification of the risk factor is not possible on an individual level and requires political, governmental, or medical interventions	There is some evidence of potential effect of intervention on the risk factor and/or outcome, but literature is controversial	>50% of moderate or high quality report an association in the same direction or less than 5 publications
Low (0)	Reproducibility and accuracy of the marker, standardization of the assay and stability of the sample are either poor or not properly documented	High and unexplained variation in a short time span and relevant effects likely to be observed between groups of thousands of individuals	Plausible hypothesis, in use as an exploratory marker, but no substantial body of evidence yet.	The risk factor is not modifiable	No intervention studies have been conducted, or no potential effect of modification has been found	No consistent effect

Table S3. Associations of each early-life risk factor with childhood obesity per critical period.					
**Author**	**Year**	**Country**	**Exposure (sub)**	**Prevalence Obesity (%)**	**Association**
**Preconception**					
**Physical**					
Maternal prepregnancy weight/BMI continuous					
*Brophy	2009[21]	UK	Maternal prepregnancy weight	5.8	Positive
*Chivers	2012[22]	Australia	Maternal prepregnancy BMI	7.8	Positive
*Dhana	2018[23]	USA	BMI	9.1	Positive
*Fernández-Barrés	2016[24]	Spain	Prepregnancy BMI	12.4	Positive
*Fisch	1975[25]	USA	Maternal height;<br>Maternal weight;<br>Maternal BMI	5.5	Positive;<br>Positive;<br>Positive
*Gittner	2013[26]	USA	Prepregnancy BMI	-	Positive
Groth	2017[27]	USA	Prepregnancy BMI	21.4	Positive
*Huang	2014[40]	USA	Prepregnancy weight	26.6	None
*Hunt	2022[28]	USA	Prepregnancy BMI	13.1	Positive
*Kjaer	2019[29]	USA	Prepregnancy BMI	37.0	Positive
*LaGasse	2011[30]	USA	Prepregnancy BMI	21.6	Positive
*Li	2011[33]	USA	Prepregnancy weight	18.0	Positive
*Mehta	2012[31]	USA	Prepregnancy BMI	-	Positive
*O'Connor	2020[32]	USA	Prepregnancy BMI	17	Positive
Ouyang	2016[34]	USA	Prepregnancy BMI	6.4	Positive
*Palma dos Reis	2022[35]	Portugal	Prepregnancy weight	?	Positive
*Rotevatn	2021[37]	Danmark	Prepregnancy BMI	2.4	Positive
*Ventura	2020[41]	USA	Prepregnancy BMI	10.9	None
*Wang	2022[36]	China	Maternal prepregnancy BMI	10.8	Positive
*Widen	2016[38]	USA	Maternal prepregnancy BMI	22	Positive
*Wojcicki	2015[42]	USA	Maternal prepregnancy BMI;	24.9	None
Zhang	2022[39]	China	Maternal prepregnancy weight	??	Positive
Maternal prepregnancy overweight/obesity					
*Flores	2013[82]	USA	Mother severely obese prepregnancy	5.7	Positive
*Gaillard	2013[197]	Netherlands	Maternal obesity prepregnancy	8.1	Positive
Hinkle	2012[201]	USA	Prepregnancy obesity	15.8	Positive
*Hu	2019[85]	USA	Prepregnancy overweight/obesity	16.6	Positive
*Janjua	2012[118]	USA	Prepregnancy BMI overweight/obese	9.6	Positive

Leonard	2017[205]	USA	Prepregnancy overweight + excessive weight gain; Prepregnancy obesity + excessive weight gain	18.0-24.0	Positive
*Navarro	2020[69]	Ireland	Prepregnancy BMI >30	8.8-29.3	Positive
*Reilly	2005[43]	UK	Maternal BMI >30 kg/m2	8.6	Positive
*Whitaker	1998[49]	USA	Maternal prepregnancy obesity	20	Positive
Paternal prepregnancy overweight/obesity					
*Reilly	2005[43]	UK	Paternal BMI >30 kg/m2	8.6	Positive
**Lifestyle**					
Smoking					
*Dhana	2018[23]	USA	Smoking	9.1	Positive
*Sharma	2008[44]	USA	Smoking	14.6	Positive
Diet					
*Dhana	2018[23]	USA	Healthy diet	9.1	None
Gete	2021[45]	Australia	Higher healthy eating index	3.3	None
Physical activity					
*Dhana	2018[23]	USA	Exercise	9.1	None
Noda	2022[46]	Japan	Maternal physical activity	9.5	None
**Pregnancy and birth**					
**Birth anthropometrics**					
Birth weight continuous					
*Brophy	2009[21]	UK	Birth weight	5.8	Positive
*Donkor	2017[62]	Norway	Birth weight	3.5	None
*Fisch	1975[25]	USA	Birth weight	5.5	Positive
*Fernández-Barrés	2016[24]	Spain	Birth weight	12.4	Positive
*Gillman	2003[106]	USA	Birth weight	6.7	Positive
*Heerman	2019[130]	USA	Birth weight	34.5	None
*Kjaer	2019[29]	USA	Birth weight	37.0	Positive
*Layte	2014[53]	Ireland	Birth weight	?	Positive
*Mardones	2008[108]	Chile	Birth weight	17.9	Positive
Mardones	2014[107]	Chile	Birth weight	16.0	Positive
*Monteiro	2003[133]	Brazil	Birth weight	7.7	None
*O'Connor	2020[32]	USA	Birth weight	17	Positive
Parker	2012[109]	USA	Birth weight	9	Positive
*Reilly	2005[43]	UK	Birth weight	8.6	Positive
*Roy	2015[167]	USA	Birth weight	17	None
*Shankaran	2010[110]	USA	Birth weight	21	Positive
*Shehadeh	2008[143]	Israel	Birth weight	-	None

*Shi	2013[60]	Canada	Birth weight	13	Positive
*Simpson	2017[134]	UK	Birth weight	4.0-6.4	None
*Skledar	2015[111]	Croatia	Birth weight	17.8	Positive
*Taveras	2009[210]	USA	Birth weight for length score	9	None
*Turner	2021[112]	UK	Birth weight	4	Positive
Vehapoglu	2017[113]	Turkey	Birth weight	7.8	Positive
*Ventura	2020[41]	USA	Birth weight	10.9	Positive
*Von Kries	2002[72]	Germany	Birth weight	2.2-8.5	Positive
*Wang	2022[36]	China	Birth weight	10.8	Positive
Zarrati	2013[128]	Iran	Birth weight	5.3	None
*Zhou	2011[104]	China	Birth weight	3.5	Positive
Birth length continuous					
*Donkor	2017[62]	Norway	Birth length	3.5	None
*Mardones	2008[108]	Chile	Birth length	17.9	Negative
Mardones	2014[107]	Chile	Birth length	16.0	None
*Shehadeh	2008[143]	Israel	Birth length	-	None
*Skledar	2015[111]	Croatia	Birth length	17.8	Positive
Low birth weight/small-for-gestational age					
Chen	2019b[126]	China	Low birth weight (<2500 g)	8.0	Positive
*Chiasson	2016[114]	USA	Low birth weight	9.9	Negative
De Sousa	2013[115]	Brazil	Low birth weight	22.0	None
*Eid	1970[129]	UK	Low birth weight	7.7	Negative
Gallo	2016[127]	Italy	Small-for-gestational age	12.7-29.1	Positive
*Goodell	2009[165]	USA	Low birth weight	18.0	None
Hack	2014[200]	USA	Extreme low birth weight	12.0-21.0	None
*Huang	2014[40]	USA	Low birth weight <2500 g	26.6	Negative
Kapral	2018[119]	USA	Low birth weight <2500 g	12.0-6.5	None
*LaGasse	2011[30]	USA	Small-for-gestational age	21.6	Negative
*Lawrence	2016[121]	USA	Low birth weight	14.3-36.4	None
*Li	2011[33]	USA	Low birth weight	18.0	Negative
*Monteiro	2003[133]	Brazil	Small-for-gestational age (<P10)	7.7	None
*Ochoa	2007[161]	Spain	Birth weight (<2800 g)	-	None
Ouyang	2016[34]	USA	Small-for-gestational age	6.4	Negative
*Reynolds	2014[59]	Ireland	Birth weight (<2500 g versus ≥2500 g)	6.6	None
*Rooney	2011[58]	USA	Birth weight < 7pounds	22.6-29.6	None
*Rotevatn	2021[37]	Denmark	Small-for-gestational age	2.4	Negative
*Sartorius	2020[124]	South Africa	Birth weight <2500 g	15.1	Negative
*Shankaran	2010[110]	USA	Intra-uterine growth restriction	21	Positive

Zarrati	2013[128]	Iran	Low birth weight (<2500 g)	5.3	Positive
High birth weight/large-for-gestational age					
*Chiasson	2016[114]	USA	High birth weight (>4000 g)	9.9	Positive
De Sousa	2013[115]	Brazil	High birth weight	22.0	Positive
Gu	2012[116]	China	Macrosomia (>4000 g)	11.4	Positive
*He	2000[117]	China	Birth weight >4000 g	-	Positive
*Huang	2014[40]	USA	High birth weight >4000 g	26.6	None
*Huus	2007[47]	Sweden	High birth BMI (>95th percentile)	4.3	Positive
*Janjua	2012[118]	USA	High birth weight (>85th percentile)	9.6	Positive
Kapral	2018[119]	USA	High birth weight >4500 g	12.0-6.5	Positive
*Kato	2014[120]	Japan	Obesity at birth	5.6-8.4	Positive
*Lawrence	2016[121]	USA	High birth weight	14.3-36.4	Positive
*Loaiza	2011[122]	Chile	Macrosomia (>4000 g)	14.6	Positive
*Mehta	2012[31]	USA	Large-for-gestational age	-	Positive
Ouyang	2016[34]	USA	Large-for-gestational age	6.4	Positive
Pan	2019[123]	China	Macrosomia	5.4	Positive
*Rooney	2011[58]	USA	Birth weight > 8.5 pounds	22.6-29.6	Positive
*Rotevatn	2021[37]	Denmark	Large-for-gestational age	2.4	Positive
*Sartorius	2020[124]	South Africa	Birth weight >4000 g	15.1	Positive
*Toschke	2002[125]	Czech Republic	Birth weight > 4000 g	3.8	Positive
Gestational age at birth continuous					
*Chen	2017[50]	Taiwan	Gestational age	14.7	None
*Heerman	2019[130]	USA	Gestational age at birth	34.5	None
*McCrory	2012[131]	Ireland	Gestational age	6.6	None
*O'Connor	2020[32]	USA	Gestational age	17	None
*Ventura	2020[41]	USA	Gestational age	10.9	None
*Mardones	2008[108]	Chile	Gestational age	17.9	Negative
Preterm birth					
Alves	2016[132]	Brazil	Preterm birth	12.7	None
*Layte	2014[53]	Ireland	Gestational age <33 wks;<br>Gestational age <36 wks	?	Positive;<br>None;
*Monteiro	2003[133]	Brazil	Gestational age (<37 wks)	7.7	None
*Reynolds	2014[59]	Ireland	Preterm birth (only ≤32 wks)	6.6	Negative
*Seipel	2013[54]	USA	Preterm birth	11-13	None
*Shankaran	2010[110]	USA	Preterm birth	21	Positive
Late-term birth					
*Chen	2017[50]	Taiwan	Late-term ≥ 42 wks	14.7	Positive
*Layte	2014[53]	Ireland	Gestational age >42 wks	?	None

Foetal growth					
Parker	2012[109]	USA	Estimated foetal growth	9	Positive
*Turner	2021[112]	UK	Estimated foetal growth	4	Positive
Placental weight					
Ouyang	2016[34]	USA	Placental weight	6.4	Positive
**Pregnancy complications**					
Mode of Delivery (caesarean section ***)***					
Carrillo-Larco	2015[91]	Peru	Mode of delivery (caesarean section)	0.7 – 1.1	Positive
*Chen	2017[50]	Taiwan	Mode of delivery (caesarean section)	14.7	Positive
Dal'Maso	2022[92]	Brazil	Mode of delivery (caesarean section)	8.3	Positive
Flemming	2013[101]	Canada	Mode of delivery (caesarean section)	9.8	None
Goldani	2013[93]	Brazil	Mode of delivery (caesarean section);<br>Mode of delivery (caesarean section)	13.0/2.1	Positive;<br>None (two cohorts)
*Hawkins	2019[66]	USA	Mode of delivery (caesarean section)	12.4 – 13.2	Positive
*Huang	2014[40]	USA	Mode of delivery (caesarean section)	26.6	None
Huh	2012[94]	USA	Mode of delivery (caesarean section)	9.2	Positive
*Lavin	2018[95]	Vietnam	Mode of delivery (caesarean section)	4.1	Positive
Masukume	2019a[102]	New Zealand	Mode of delivery (caesarean section)	8.6	None
Mueller	2015[96]	USA	Mode of delivery (caesarean section)	25.2	Positive
Pei	2014[97]	Germany	Mode of delivery (caesarean section)	11	Positive (only 2 y)
Ralphs	2021[103]	UK	Mode of delivery (caesarean section)	5.2	None
Rifas-Shiman	2021[105]	Belarus	Mode of delivery (caesarean section)	3.8 – 5.9	None
*Rooney	2011[58]	USA	Mode of delivery (caesarean section)	22.6-29.6	Positive
*Rotevatn	2021[37]	Danmark	Mode of delivery (caesarean section)	2.4	Positive
*Seipel	2013[54]	USA	Mode of delivery (caesarean section)	11-13	None
Si	2022[98]	China	Mode of delivery (caesarean section)	3.8	Positive
Sitarik	2020[99]	USA	Mode of delivery (planned caesarean section)	20.3	Positive
Yuan	2016[100]	USA	Mode of delivery (caesarean section)	13	Positive
*Zhou	2011[104]	China	Mode of delivery (caesarean section)	3.5	None
Gestational diabetes					
*Chen	2017[50]	Taiwan	Gestational diabetes	14.7	Positive
*Fernández-Barrés	2016[24]	Spain	Gestational diabetes	12.4	None
*Flores	2013[82]	USA	Gestational diabetes	5.7	Positive
*Gillman	2003[106]	USA	Gestational diabetes	6.7	None
Hakanen	2016[83]	Finland	Gestational diabetes	3.0-12.8	Positive
*Hawkins	2019[66]	USA	Gestational diabetes	12.4 – 13.2	Positive

Herath	2020[84]	Sri Lanka	Hyperglycaemia in pregnancy	3.2 – 5.7	Positive
*Hu	2019[85]	USA	Gestational diabetes	16.6	Positive
Lowe	2018[86]	USA	Gestational diabetes	8.2	Positive
Lowe	2019[87]	Thailand;<br>Barbados; USA;<br>UK; China;<br>Israel; Canada	Fasting glucose;<br>1 h glucose;<br>2 h glucose;<br>HbA1c	12.3	Positive;<br>Positive;<br>Positive;<br>Positive
*Mehta	2012[31]	USA	Gestational diabetes	?	None
Nehring	2013[88]	Germany	Gestational diabetes	2.6	Positive
Ouyang	2016[34]	USA	Gestational diabetes	6.4	Positive
Pettitt	1983[89]	USA	Gestational diabetes	-	Positive
Pettitt	1998[90]	USA	Glucose during pregnancy	-	Positive (at 5-9 y)
Pitchika	2018[209]	USA, Finland,<br>Germany,<br>Sweden	Gestational diabetes;<br>Diabetes type 1<br>Diabetes type 2	5.7	None;<br>Negative (only 6 y)<br>None
*Roy	2015[167]	USA	Gestational diabetes	17	None
Thaware	2015[211]	UK	Gestational diabetes	12.7	None
*Whitaker	1998[49]	USA	Gestational Diabetes	20	None
Wright	2009[216]	USA	Gestational Diabetes	9.3	None
Wroblewska-Senuik	2009[217]	Poland	Gestational Diabetes	13	None
Preeclampsia					
*Palma dos Reis	2022[35]	Portugal	Preeclampsia	?	None
Infection					
Li	2020[207]	USA	Infection without antibiotic use vs control	16.2-21.1	Positive
Antibiotic use					
Li	2020[207]	USA	Antibiotic use (with or without infection)	16.2-21.1	None
Margetaki	2022[180]	Greece	Prenatal antibiotics use	7.8-12.0	None (4 y);<br>Positive (6 y)
Mueller	2015[96]	USA	Use of antibiotics	25.2	Positive
Sakurai	2022[179]	Japan	Antibiotic use (in 2nd or 3rd trimester)	9.4	Positive (only in females)
Wang	2018[181]	USA	Antibiotic use	8.9 (4y)<br>4.6 (7y)	None (4y)<br>Positive (7y, 2nd trimester)
**Cord blood biomarkers**					
*Donahue	2011[75]	USA	Higher DHA+EPA/n-3 fatty acids;<br>Higher n-6:n-3 ratio/AA:DHA + EPA ratio/LA:ALA ratio	9.4	Negative;<br>Positive
*Simpson	2017[134]	UK	Leptin<br>Adiponectin	4.0-6.4	None

Sorrow	2019[135]	USA	Metabolomics	-	Positive/negative
**Sociodemographic**					
Maternal age					
*Chen	2017[50]	Taiwan	Age mother at birth	14.7	None
*Donkor	2017[62]	Norway	Age mother at birth	3.5	None
*Fernández-Barrés	2016[24]	Spain	Maternal age at birth	12.4	None
*Fisch	1975[25]	USA	Age mother at birth	5.5	Positive
*Flores	2013[82]	USA	Maternal age at birth	5.7	None
*Gittner	2013[26]	USA	Age	-	None
*Huang	2014[40]	USA	Age	26.6	None
*Hunt	2022[28]	USA	Age	13.1	Negative
*Huus	2007[47]	Sweden	Maternal age	4.3	None
*Kjaer	2019[29]	USA	Age	37.0	None
*Layte	2014[53]	Ireland	Age	?	None
*Seipel	2013[54]	USA	Maternal age at birth	11-13	Positive
*Ventura	2020[41]	USA	Maternal age	10.9	None
*Wallby	2017[154]	Sweden	Maternal age	1.9	None
*Wojcicki	2015[42]	USA	Maternal age	24.9	None
Paternal age					
*Fisch	1975[25]	USA	Age father at birth	5.5	Positive
*Huus	2007[47]	Sweden	Paternal age	4.3	None
Maternal education					
*Chivers	2012[22]	Australia	Education (lower)	7.8	Positive
*Fernández-Barrés	2016[24]	Spain	Maternal educational level	12.4	None
*Huang	2014[40]	USA	Education (lower)	26.6	None
*Hunt	2022[28]	USA	Education	13.1	None
*Kjaer	2019[29]	USA	Education	37.0	None
*Loaiza	2011[122]	Chile	Finished elementary school	14.6	Negative
*Mardones	2008[108]	Chile	Education (>8 years)	17.9	Positive
*Rotevatn	2021[37]	Denmark	Maternal education (higher)	2.4	Negative
*Seipel	2013[54]	USA	Maternal education (higher)	11-13	Negative
*Shankaran	2010[110]	USA	Maternal education (higher)	21	Positive
*Ventura	2020[41]	USA	Maternal education (higher)	10.9	None
*Wallby	2017[154]	Sweden	Maternal educational level (lower)	1.9	Positive
*Wen	2022[81]	USA	Maternal education (lower)	14.9	Positive
*Wojcicki	2015[42]	USA	Maternal education (higher)	24.9	Positive
Paternal education					
*Wojcicki	2015[42]	USA	Paternal education (lower)	24.9	Positive

Income					
*Rotevatn	2021[37]	Denmark	Household income (higher)	2.4	Negative
*Ventura	2020[41]	USA	Income	10.9	None
*Wen	2022[81]	USA	Family income (lower)	14.9	Positive
*Wojcicki	2015[42]	USA	Income (lower)	24.9	Positive
Maternal social economic status					
*Fernández-Barrés	2016[24]	Spain	Social class	12.4	None
*Fisch	1975[25]	USA	Socioeconomic index (higher)	5.5	None
*Ingstrup	2012[204]	Denmark	Socioeconomic status (lower)	9.9	Positive
*Layte	2014[53]	Ireland	Social class	?	None
Maternal ethnicity					
*Layte	2014[53]	Ireland	Non-white (African) versus white (Irish, UK, European)	?	None
*Shankaran	2010[110]	USA	Ethnicity (non-white)	21	Negative
*Ventura	2020[41]	USA	Ethnicity (other than white)	10.9	None
*Wojcicki	2015[42]	USA	Ethnicity (non-White)	24.9	Positive;
Parity					
*Frondelius	2018[64]	Sweden	Parity	3.0	None
*Gittner	2013[26]	USA	Parity	-	Negative
*Layte	2014[53]	Ireland	Parity	?	None
*Rotevatn	2021[37]	Denmark	Parity	2.4	Positive
*Ventura	2020[41]	USA	Parity	10.9	None
*Wallby	2017[154]	Sweden	Parity	1.9	None
*Huus	2007[47]	Sweden	Parity (any siblings)	4.3	None
*Lavin	2018[95]	Vietnam	Multiparous	4.1	Negative
Maternal employment					
*Gittner	2013[26]	USA	Employment (yes)	-	Positive
Maternal relationship status					
*Huus	2007[47]	Sweden	Maternal relationship status (single)	4.3	None
*Ventura	2020[41]	USA	Marital status	10.9	None
*Wojcicki	2015[42]	USA	Marital status mother (not married)	24.9	Positive
**Lifestyle**					
Maternal smoking					
Chen	2006[61]	USA	Smoking	4.3 – 8.6	Positive
*Chivers	2012[22]	Australia	Smoking	7.8	Positive
*Donkor	2017[62]	Norway	Smoking	3.5	Positive;
Durmus	2011[63]	Netherlands	Smoking	2.3	Positive
*Fernández-Barrés	2016[24]	Spain	Smoking	12.4	None

*Fisch	1975[25]	USA	Smoking	5.5	None
*Frondelius	2018[64]	Sweden	Smoking	3.0	Positive
Harris	2013[65]	USA	Smoking	18.9	Positive
*Hawkins	2019[66]	USA	Smoking	12.4 – 13.2	Positive
Horiuchi	2021[48]	Japan	Maternal smoking	-	None
*Huang	2014[40]	USA	Smoking	26.6	Positive
Ino	2011[67]	Japan	Smoking	6.0-16.0	Positive
*Janjua	2012[118]	USA	Smoking	9.6	None
*Layte	2014[53]	Ireland	Smoking	?	Positive
Mizutani	2007[68]	Japan	Smoking	2.7	Positive
*Navarro	2020[69]	Ireland	Smoking	8.8-29.3	Positive (for 5y)
*O'Connor	2020[32]	USA	Smoking	17	Positive
*Palma dos Reis	2022[35]	Portugal	Smoking	?	Positive
Power	2002[70]	UK	Smoking	-	Positive (only for 16y)
*Reilly	2005[43]	UK	Smoking	8.6	Positive
*Reynolds	2014[59]	Ireland	Smoking	6.6	Positive
*Rooney	2011[58]	USA	Smoking	22.6-29.6	Positive
*Rotevatn	2021[37]	Danmark	Smoking	2.4	Positive
*Sharma	2008[44]	USA	Smoking	14.6	Positive
*Seipel	2013[54]	USA	Smoking	11-13	Positive
*Shankaran	2010[110]	USA	Smoking	21	None
*Shi	2013[60]	Canada	Smoking	13	Positive
Suzuki	2009[71]	Japan	Smoking	3	Positive
*Von Kries	2002[72]	Germany	Smoking	2.2-8.5	Positive
Wang	2014[73]	USA	Smoking	23.2	Positive
*Wojcicki	2015[42]	USA	Smoking	24.9	None
Paternal smoking					
Horiuchi	2021[48]	Japan	Paternal smoking	-	None
Maternal alcohol					
*Huang	2014[40]	USA	Alcohol use	26.6	Negative
*LaGasse	2011[30]	USA	Alcohol	21.6	None
*Layte	2014[53]	Ireland	Alcohol use (light)	?	Negative
*Navarro	2020[69]	Ireland	Alcohol use	8.8-29.3	None
*Seipel	2013[54]	USA	Alcohol use	11-13	Positive
*Shankaran	2010[110]	USA	Alcohol use	21	None
Maternal drugs					
*Seipel	2013[54]	USA	Drugs use	11-13	None
*Shankaran	2010[110]	USA	Drugs use	21	None

*LaGasse	2011[30]	USA	Cocaine	21.6	Positive
Diet					
*Fernández-Barrés	2016[24]	Spain	Maternal caloric intake<br>Mediterranean diet	12.4	None<br>None
*Callanan	2021[80]	Ireland	Low GI diet	6.5	None
Chen	2019a[74]	Ireland	Diet, higher GI;<br>Diet, higher GL (glycaemic load);<br>Diet, insulinemic index (II)	5.0	None
*Donahue	2011[75]	USA	Diet, more fish/fatty acid/DHA+EPA intake	9.4	Negative
Hakola	2017[76]	Finland	Fatty acid intake	2.0 - 10.0	Positive, negative and none
Hu	2020[77]	USA	Diet, fast food pattern;<br>Diet, processed food pattern	16.4	Positive;<br>None
Kadawathagedara	2018[78]	Norway	Acrylamide intake	0.4-1.6	Positive
Klebanoff	2015[79]	USA	Serum paraxantine	6.5	None
Mizutani	2007[68]	Japan	Maternal breakfast habit (skipping)	2.7	Positive
*Navarro	2020[69]	Ireland	Maternal diet	8.8-29.3	None
Suzuki	2009[71]	Japan	Diet (skipping breakfast)	3	Positive
Physical activity					
*Fernández-Barrés	2016[24]	Spain	Physical activity	12.4	None
*Navarro	2020[69]	Ireland	Maternal physical activity	8.8-29.3	None
Psychological stress and disorders					
Bryl	2022[194]	Poland	Maternal stress	5.0	None
*Ingstrup	2012[204]	Denmark	Maternal distress;<br>Anxious;<br>Depressed;<br>Stressed;<br>Worried;<br>Lack of support	9.9	None;<br>None;<br>None;<br>None;<br>Positive;<br>None
*Kjaer	2019[29]	USA	Depression	37.0	Negative
Caffeine					
Li	2015[206]	USA	Caffeine	?	Positive
Sleep duration					
Mizutani	2007[68]	Japan	Maternal sleep duration >8h	2.7	Negative
Suzuki	2009[71]	Japan	Sleep duration (longer)	3	Negative
Fetal musical education					
*Zhou	2011[104]	China	Fetal musical education (lower)	3.5	Positive
**Physical**					

Paternal BMI or overweight/obesity					
*Chivers	2012[22]	Australia	Paternal BMI at birth	7.8	Positive
*Whitaker	1998[49]	USA	Paternal obesity	20	Positive
Maternal BMI or ovesrweight/obesity					
*Lavin	2018[95]	Vietnam	Maternal overweight/obesity	4.1	Positive
*Wallby	2017[154]	Sweden	Maternal BMI;<br>Maternal overweight/obesity;	1.9	Positive<br>Positive
Gestational weight gain continuous					
*Chen	2017[50]	Taiwan	Gestational weight gain	14.7	Positive
Diesel	2015b[51]	USA	Gestational weight gain	21.0	Positive
*Fernández-Barrés	2016[24]	Spain	Gestational weight gain	12.4	None
*Fisch	1975[25]	USA	Gestational weight gain	5.5	None
Groth	2017[27]	USA	Gestational weight gain	21.4	Positive
Hivert	2016[52]	USA	Gestational weight gain	12.0	Positive
*Hunt	2022[28]	USA	Gestational weight gain	13.1	Positive
*Layte	2014[53]	Ireland	Gestational weight gain	?	Positive
*Li	2011[33]	USA	Gestational weight gain	18.0	Positive
*Seipel	2013[54]	USA	Gestational weight gain	11-13	Positive (only 10y)
Oken	2007[55]	USA	Gestational weight gain	9	Positive
Oken	2008[56]	USA	Gestational weight gain	6.5	Positive
Oken	2009[57]	USA	Gestational weight gain	10	Positive (only women with prepregnancy BMI 25-30)
*Wang	2022[36]	China	Gestational weight gain	10.8	Positive
*Rooney	2011[58]	USA	Gestational weight gain	22.6-29.6	Positive
Widen	2016[38]	USA	Gestational weight gain	22	Positive
Excessive gestational weight gain					
Diesel	2015a[195]	USA	Excessive gestational weight gain	16.4	Positive
Ensenauer	2013[196]	Germany	Excessive gestational weight gain	2.4	Positive
Hu	2019[85]	USA	Excessive gestational weight gain	16.6	Positive
Liu	2019[208]	USA	Excessive gestational weight gain	11.0	None
Ouyang	2016[34]	USA	Gestational weight gain (excessive)	6.4	Positive
Inadequate gestational weight gain					
*Liu	2019[208]	USA	Inadequate gestational weight gain	11.0	None
*Seipel	2013[54]	USA	Gestational weight loss	11-13	Positive (only at 10y)
Ouyang	2016[34]	USA	Gestational weight gain (inadequate)	6.4	Negative
Maternal lipids (standard pregnancy screening)					
Thaware	2018[212]	UK	Lipids	11.1	None

**Environmental**					
Guo	2020a[198]	China	Urinary bisphenol A	15.8	Positive
Guo	2020b[199]	China	Polybrominated diphenyl ethers (6 metabolites)	8.8	5 None, 1 negative (BDE-154)
Vafeiadi	2015[213]	Greece	Persistent organic pollutants	6.9	Positive
Vafeiadi	2016[214]	Greece	Bisphenol A exposure	13.3	Positive
Warner	2013[215]	USA	Pesticide exposure (Dichlorodiphenyltrichloroethane& Dichlorodiphenyldichloroethylene)	35.6	None
**Infancy**					
**Feeding patterns**					
Early solid food introduction					
*Brophy	2009[21]	UK	Early solid food introduction (<3 months)	5.8	Positive
*Gittner	2013[26]	USA	Timing of solid food introduction	-	None
Gooze	2011[157]	USA	Timing of solid food introduction <3 months	17.6	Negative
Huh	2011[202]	USA	Timing of solid food introduction (among breastfed; <4mnth)<br>Timing of solid food introduction (among formulafed; <4mnth)	9.0	None<br><br>Positive
Moss	2014[140]	USA	Timing of solid food introduction	14.3	None
*Reilly	2005[43]	UK	Introduction to solid food	8.6	None
*Ventura	2020[41]	USA	Introduction to complementary foods 4- <6 months<br>Introduction to complementary foods > 6 months	10.9	None
*Von Kries	2002[72]	Germany	Introduction to solid foods < 4 months	2.2-8.5	None
*Zhou	2011[104]	China	Solid food introduction (<4 mnth)	3.5	Positive
Breastfeeding (yes/no)					
*Chiasson	2016[114]	USA	Breastfeeding (partial or exclusive)	9.9	Negative
Ehrenthal	2016[136]	USA	Breastfeeding	13.4	Negative
*Fernández-Barrés	2016[24]	Spain	Breastfeeding	12.4	None
*Gittner	2013[26]	USA	Breastfeeding	-	None
Gooze	2011[157]	USA	Breastfeeding	17.6	Positive
*Hawkins	2019[66]	USA	Breastfeeding	12.4 – 13.2	Negative
*Heerman	2019[130]	USA	Breastfeeding	34.5	None
Hildebrand	2022[137]	USA	Breastfeeding	13.0	Negative
*Huang	2014[40]	USA	Breastfeeding	26.6	Negative
*Hunt	2022[28]	USA	Exclusive breastfeeding 6 months vs not exclusively breastfed for 6 months	13.1	Negative
Huus	2008[203]	Sweden	Exclusive breastfeeding < 4months vs not exclusively breastfed for 4 months.	4.3	None

Jing	2014[159]	China	Breastfeeding	4.0	None
Jwa	2014[138]	Japan	Exclusive breastfeeding vs formula feeding	1.6 – 2.4	Negative
*Kjaer	2019[29]	USA	Exclusive breastfeeding at 4-6 wks	37.0	None
*Li	2011[33]	USA	Breastfeeding	18.0	Negative
Mayer-Davis	2006[150]	USA	Breastfeeding	6.7	None
Metzger	2010[139]	USA	Breastfeeding	-	Negative
Michels	2007[160]	USA	Breastfeeding	21.9	None
Moss	2014[140]	USA	Breastfeeding	14.3	Negative
*O'Connor	2020[32]	USA	Breastfeeding until 6 months (vs none)	17	None
Park	2015[141]	Korea	Mixed feeding vs predominantly breast fed	9.8	Positive
Pattison	2019[142]	USA	Breastfeeding	10.0	Negative
*Reilly	2005[43]	UK	Breastfeeding	8.6	None
*Seipel	2013[54]	USA	Breastfeeding	11-13	Negative
*Shehadeh	2008[143]	Israel	Breastfeeding (vs combined and formula)	-	Negative
*Shi	2013[60]	Canada	Exclusive breastfeeding for 6 months vs no breastfeeding	13	Negative
*Toschke	2002[125]	Czech Republic	Breastfeeding	3.8	Negative
Van Rossem	2011[144]	USA	Breastfeeding	3.4-9.2	Negative
*Von Kries	2002[72]	Germany	Breastfeeding	2.2-8.5	Negative
*Wang	2017[145]	USA	Breastfeeding at 1 month	18.4	Negative
Yamakawa	2013[146]	Japan	Partial or exclusive breastfeeding	2	Negative
Breastfeeding (duration)					
Burdette	2007[147]	USA	Breastfeeding > 4months	18	Negative
*Donkor	2017[62]	Norway	Breastfeeding duration < 4 months	3.5	Positive
*Fernández-Barrés	2016[24]	Spain	Breastfeeding duration	12.4	None
*Frondelius	2018[64]	Sweden	Breastfeeding duration	3.0	None
Gooze	2011[157]	USA	Breastfeeding duration	17.6	None
Grube	2015[148]	Germany	Breastfeeding duration >4 months	6.4	Negative
*Heerman	2019[130]	USA	Breastfeeding duration	34.5	None
Hildebrand	2022[137]	USA	Breastfeeding duration >6 months	13.0	Negative
Hummel	2021[149]	USA; Finland; Germany; Sweden	Any breastfeeding >6 months;<br>Exclusive breastfeeding >3 months	5.3	Negative;<br>Negative
Izadi	2013[158]	Iran	Breastfeeding duration	?	None
Jing	2014[159]	China	Breastfeeding duration	4.0	None
*Layte	2014[53]	Ireland	Breastfeeding duration >6 months	?	Negative
Martin	2013[155]	Belarus	Breastfeeding duration	5.2	None
Martin	2017[156]	Belarus	Exclusive Breastfeeding > 3 months	4.4	None
Mayer-Davis	2006[150]	USA	Breastfeeding duration > 9 months	6.7	Negative
*McCrory	2012[131]	Ireland	Breastfeeding duration >13 weeks	6.6	Negative

Michels	2007[160]	USA	Breastfeeding duration	21.9	None
Morovic	2019[151]	Croatia	Breastfeeding < 6 months (vs >6 months)	12.2	Positive
*Ochoa	2007[161]	Spain	Breast feeding > 3 months	-	None
Pattison	2019[142]	USA	Breastfeeding duration	10.0	None
*Reynolds	2014[59]	Ireland	Breastfeeding >11 weeks	6.6	Negative
Shields	2006[162]	Australia	Breastfeeding duration	4.6-6.9	None
*Skledar	2015[111]	Croatia	Breastfeeding duration	17.8	None
Tambalis	2018[152]	Greece	Breastfeeding duration >1 month	?	Negative
Toschke	2007[153]	UK	Breastfeeding duration	6.0	Negative
Van Rossem	2011[144]	USA	Breastfeeding duration	3.4-9.2	Negative
*Ventura	2020[41]	USA	Breastfeeding duration >16 weeks	10.9	None
*Wallby	2017[154]	Sweden	Breastfeeding duration	1.9	Negative
*Wang	2017[145]	USA	Breastfeeding > 6 months	18.4	None
*Wojcicki	2015[42]	USA	Breastfeeding (in months continuously)	24.9	Negative
Milk formula type					
Jwa	2014[138]	Japan	Mixed feeding vs formula feeding	1.6 – 2.4	Negative
Mayer-Davis	2006[150]	USA	Breastfeeding; predominantly breast; predominantly formula; formula only	6.7	None
Weber	2014[183]	Belgium; Germany; Italy; Poland; Spain	High protein formula vs low-protein formula	6.4	Positive
**Infant anthropometrics**					
BMI/weight					
*Huus	2007[47]	Sweden	High BMI 1 year (>95th percentile)	4.3	Positive
*Taveras	2009[210]	USA	Weight for length at 6 months	9	Positive
BMI/weight change					
Aris	2017[163]	Singapore	BMI change	7.5	Positive
Aris	2018[164]	USA; Belarus	Weight change	USA: 12.3; Belarus: 5.0	Positive
*Eid	1970[129]	UK	Excessive postnatal weight gain	7.7	Positive
*Goodell	2009[165]	USA	Rapid postnatal weight gain	18.0	Positive
*Kato	2014[120]	Japan	Height change; Weight change	5.6-8.4	None; Positive
*LaGasse	2011[30]	USA	Early weight gain	21.6	Positive
*Li	2011[33]	USA	Rapid infant weight gain	18.0	Positive
Liu	2017[166]	USA	Infant BMI trajectory: high as compared to low	11.0	Positive
*Monteiro	2003[133]	Brazil	Weight/height gain in first 24 months	7.7	Positive
*O'Connor	2020[32]	USA	Weight gain in first 24 months	17	Positive
*Rooney	2011[58]	USA	Weight gain in first 4 months	22.6-29.6	Positive

*Rotevatn	2021[37]	Danmark	Infant weight gain (rapid)	2.4	Positive
*Roy	2015[167]	USA	>= 90th percentile of weight checks	17	Positive
Smego	2017[168]	USA	Weight for length in first 24 months	4.5-12	Positive
*Taveras	2011[169]	USA	Number of crossed major weight-for-length percentiles	13.9	Positive
*Ventura	2020[41]	USA	Weight for age in first year	10.9	Positive
NICU admission					
*Reynolds	2014[59]	Ireland	NICU admission	6.6	None

* When studies examined more than one risk factor, findings for each risk factor were assessed and presented separately.
For presentation purposes, directions of effects were harmonized between studies to present summarized results.

Figure S1-3. Bias assessment of the included studies.

Figure 1a. Risk of Bias assessment. Preconception Case-Control studies

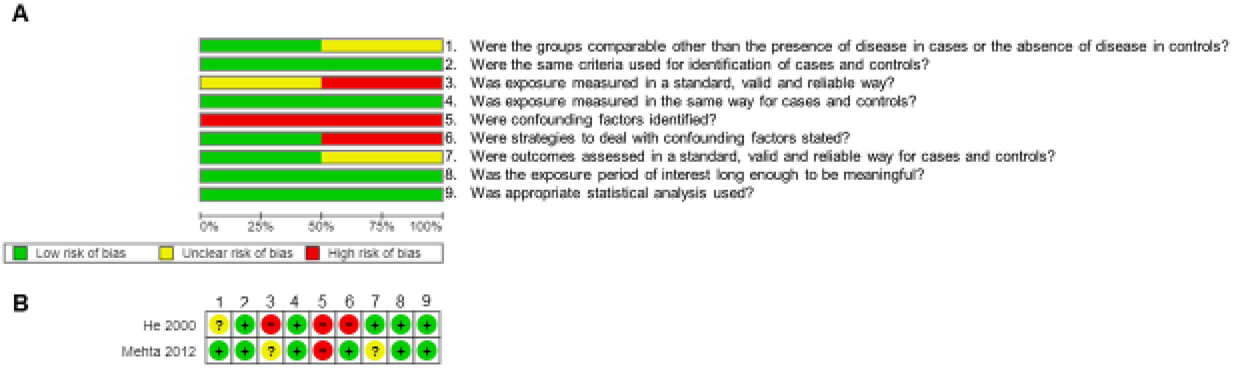

Figure 1b. Risk of Bias assessment. Preconception Prospective cohort studies

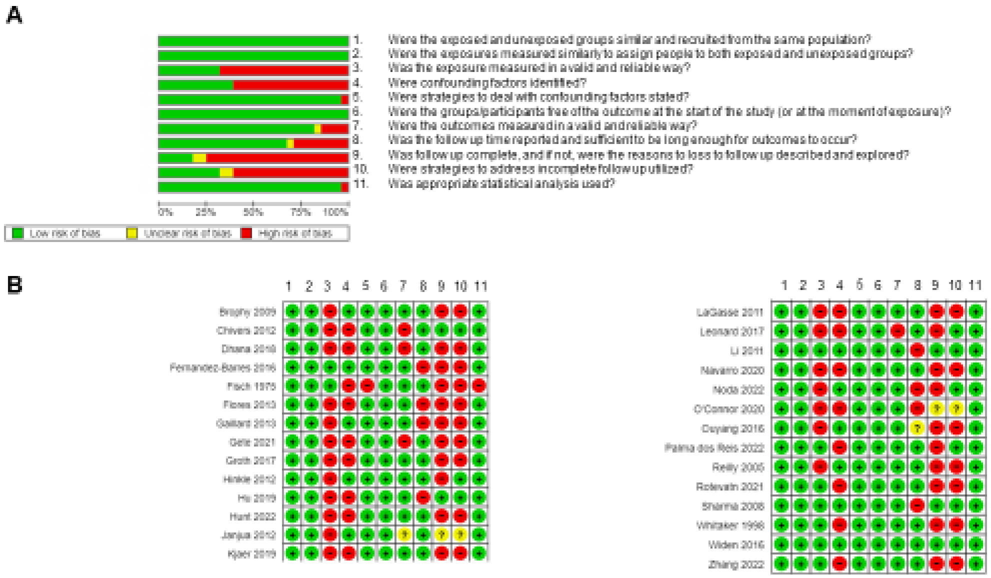

Figure 1c. Risk of Assessment. Preconception Retrospective cohort studies

A

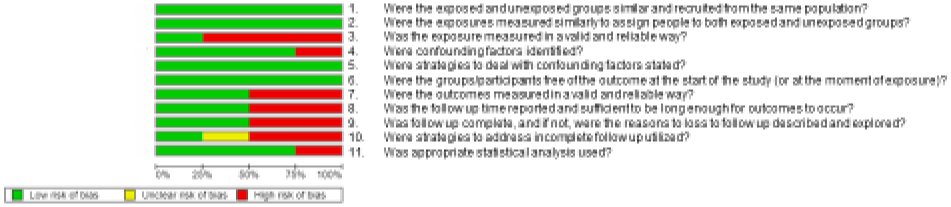

B

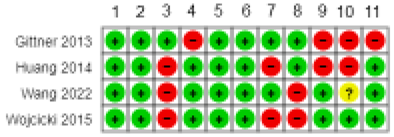

Figure 2a. Risk of Bias Assessment. Pregnancy and birth Case-Control studies

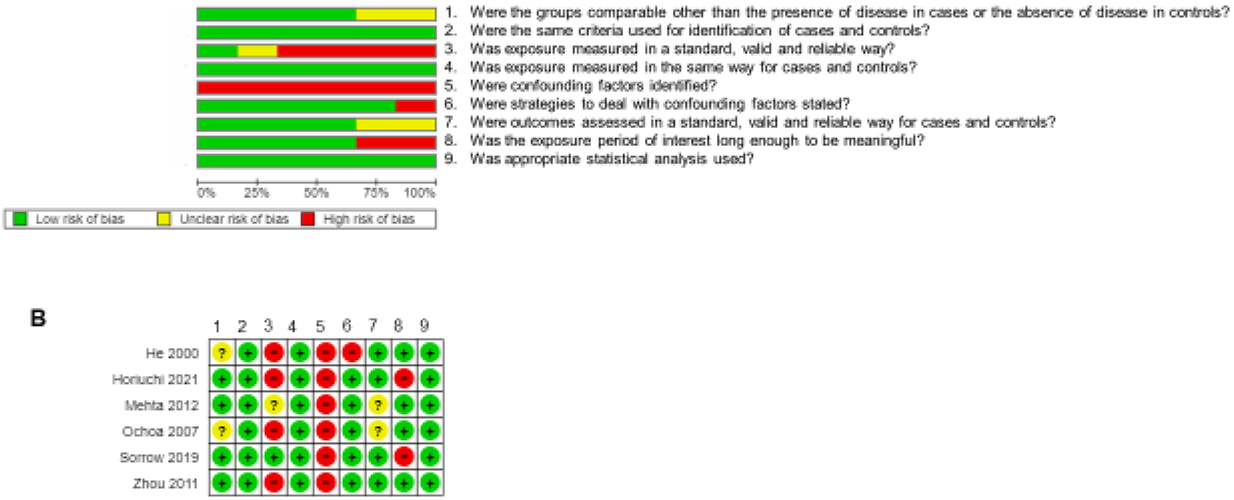

Figure 2b. Risk of Bias Assessment.
Pregnancy and birth RCT studies

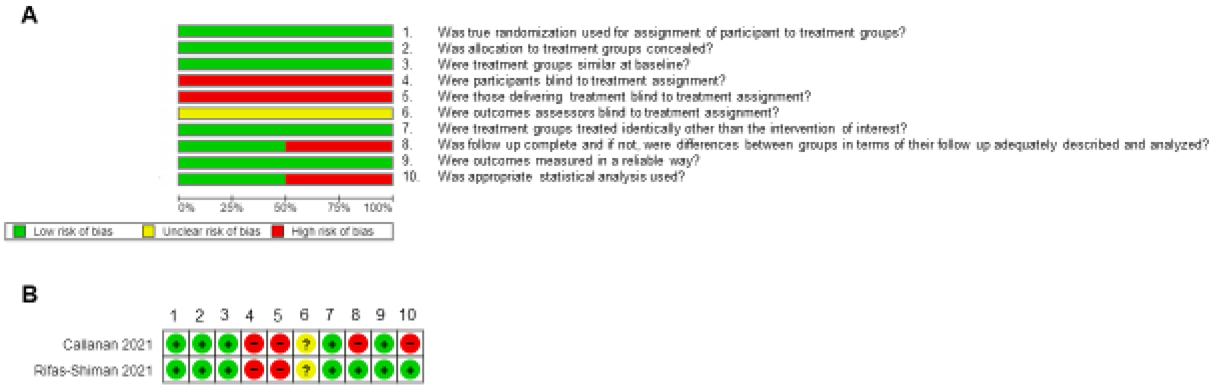

Figure 2c. Risk of Bias Assessment. Pregnancy and birth Prospective cohort studies

A

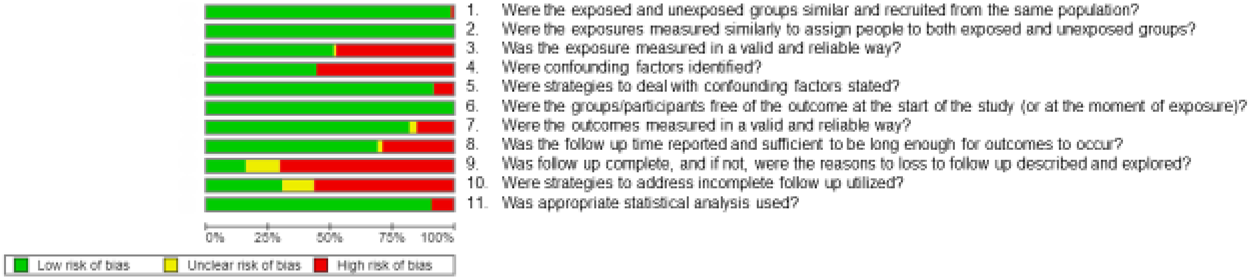

Figure 2c. Risk of Bias Assessment. Pregnancy and birth Prospective cohort studies

CONTINUED

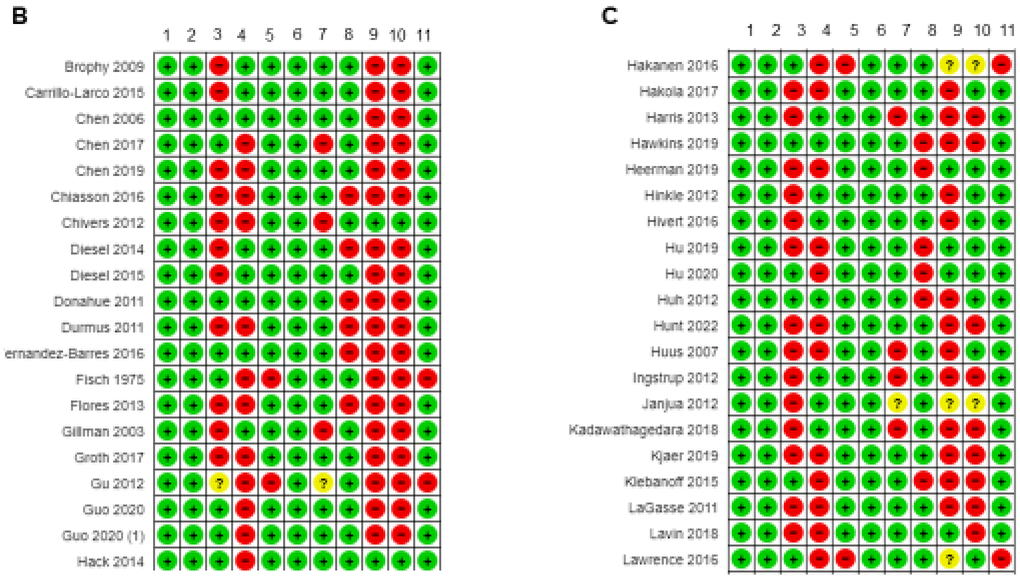

Figure 2c. Risk of Bias Assessment. Pregnancy and birth Prospective cohort studies

CONTINUED

D

	1	2	3	4	5	6	7	8	9	10	11
Layte 2014	+	+	+	+	+	+	+	−	+	+	+
Leonard 2017	+	+	−	−	+	+	−	+	−	+	+
Li 2011	+	+	+	+	+	+	+	−	+	+	+
Li 2015	+	+	+	+	+	+	+	+	?	?	+
Li 2020	+	+	+	+	+	+	+	?	?	?	+
Liu 2017	+	+	−	+	+	+	−	+	−	−	+
Liu 2019	+	+	−	+	+	+	−	+	−	−	+
Loaiza 2011	+	+	+	−	+	+	?	+	?	?	+
Lowe 2018	+	+	+	−	+	+	+	+	−	−	+
Lowe 2019	+	+	+	−	+	+	+	+	−	−	+
Mardones 2008	+	+	+	−	+	+	+	+	+	+	+
Margetaki 2022	+	+	−	+	+	+	+	+	−	−	+
Masukume 2019	+	+	+	+	+	+	+	−	?	+	+
Mizutani 2007	+	+	−	−	+	+	+	+	−	−	+
Monteiro 2003	+	+	−	−	+	+	+	+	−	+	+
Mueller 2015	+	+	−	−	+	+	+	+	+	+	+
Navarro 2020	+	+	−	−	+	+	+	+	−	−	+
O'Connor 2020	+	+	−	−	+	+	+	−	?	?	+
Oken 2007	+	+	−	+	+	+	+	−	−	+	+

E

	1	2	3	4	5	6	7	8	9	10	11
Oken 2008	+	+	−	−	+	+	−	+	−	+	+
Oken 2009	+	+	−	−	+	+	+	−	−	+	+
Ouyang 2016	+	+	−	+	+	+	+	?	−	−	+
Palma dos Reis 2022	+	+	+	−	+	+	+	+	−	+	+
Parker 2012	+	+	+	−	+	+	+	−	−	+	+
Pei 2014	+	+	−	−	+	+	+	+	−	+	+
Pettitt 1983	+	+	+	−	−	+	+	+	?	?	−
Pettitt 1998	+	+	+	−	−	+	+	+	?	?	−
Pitchika 2018	−	+	+	−	+	+	+	+	−	−	+
Power 2002	+	+	−	−	−	+	+	+	−	−	−
Ralphs 2021	+	+	+	+	+	+	+	−	−	−	+
Reilly 2005	+	+	−	+	+	+	+	+	−	−	+
Rooney 2011	+	+	+	−	+	+	+	+	−	+	+
Rotevatn 2021	+	+	+	−	+	+	+	+	−	−	+
Roy 2015	+	+	+	−	+	+	+	−	?	?	+
Sakurai 2022	+	+	+	−	+	+	+	−	−	−	+
Seipel 2013	+	+	−	+	+	+	−	+	−	−	+
Shankaran 2010	+	+	+	+	+	+	+	+	−	−	+
Sharma 2008	+	+	+	+	+	+	+	−	+	+	+
Si 2022	+	+	+	−	+	+	+	−	−	−	+

Figure 2c. Risk of Bias Assessment. Pregnancy and birth Prospective cohort studies

CONTINUED

F

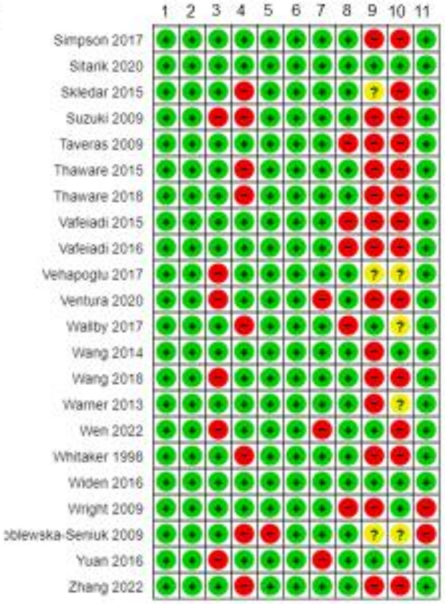

Figure 2d. Risk of Bias Assessment. Pregnancy and birth Retrospective cohort studies

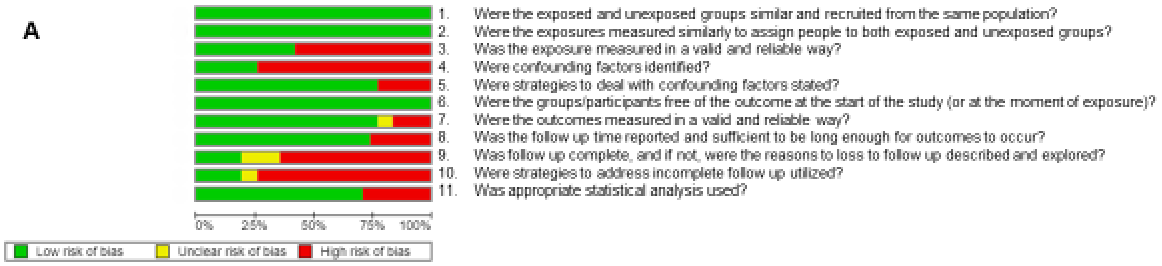

Figure 2e. Risk of Bias Assessment. Pregnancy and birth Retrospective cohort studies

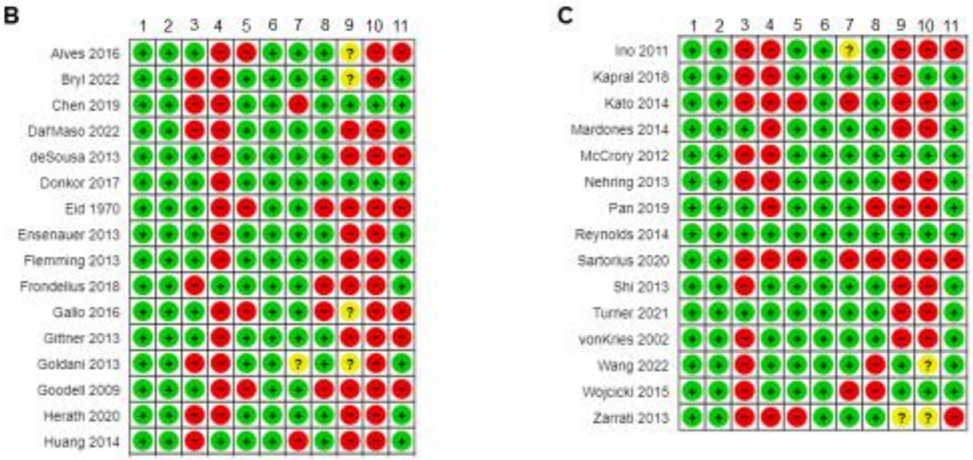

Figure 3b. Risk of Bias Assessment. Early Infancy RCT studies

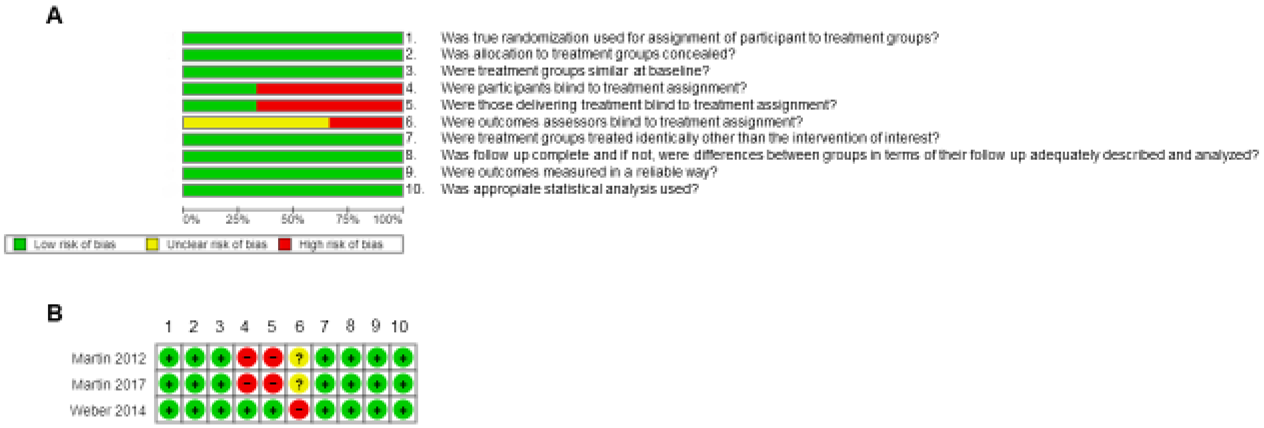

Figure 3c. Risk of Bias Assessment. Early Infancy Prospective cohort studies

CONTINUED

A

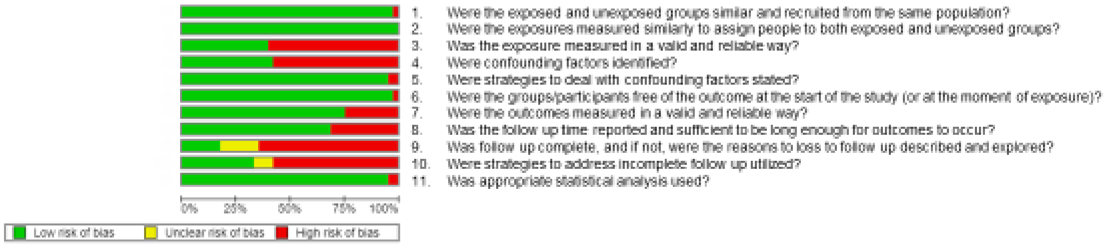

Figure 3c. Risk of Bias Assessment. Early Infancy Prospective cohort studies

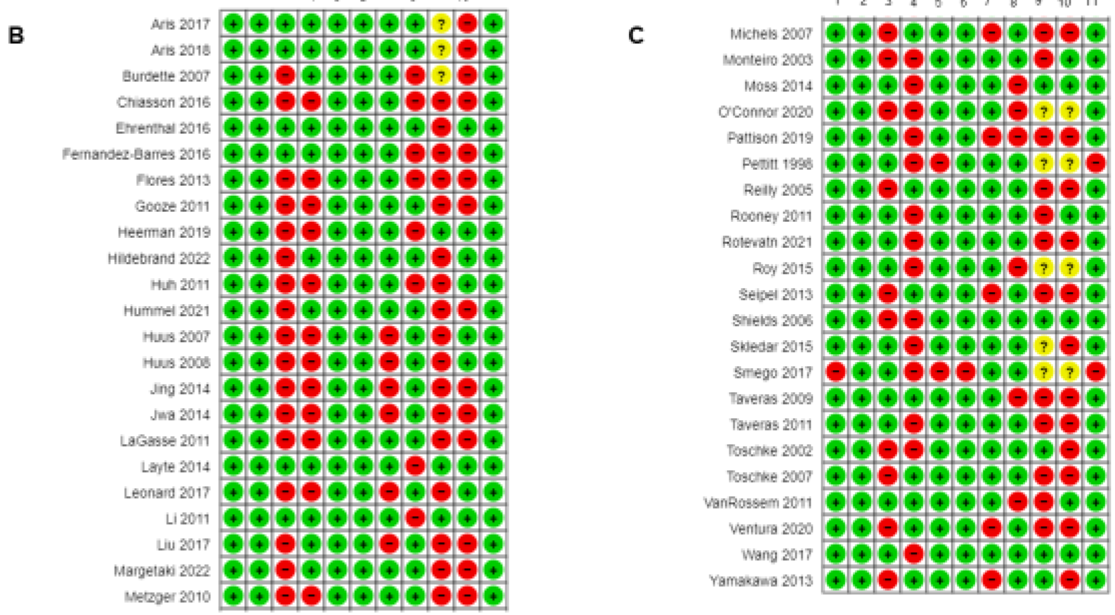

Figure 3d. Risk of Bias graph and summary of Early Infancy Retrospective cohort studies

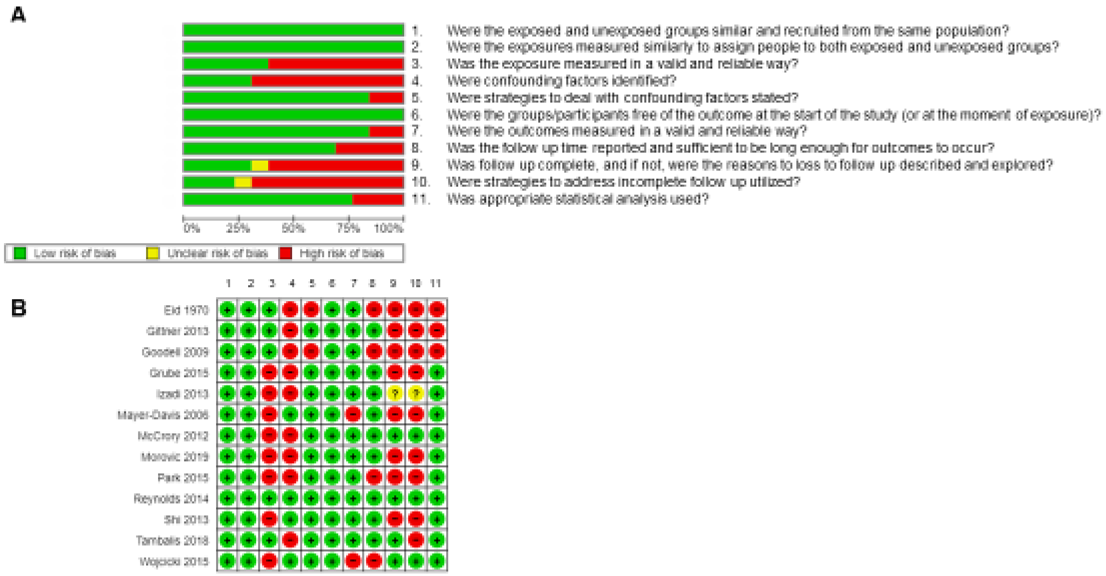

Figure S4. Quality assessment of the 23 consistently associated risk factors with childhood obesity*.

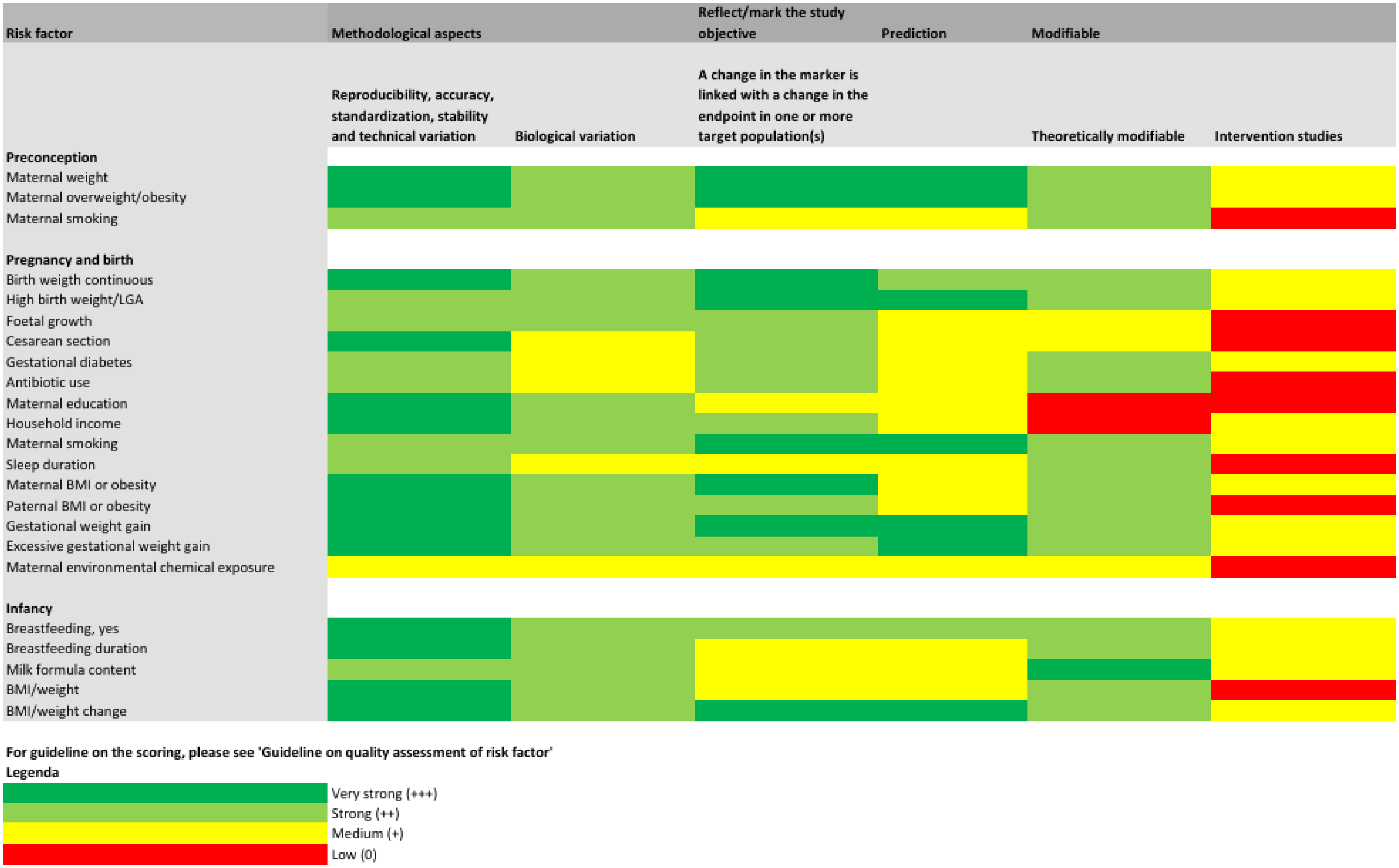

*Scores based on table S2
